# Deep Learning-Based Multiple Sclerosis Lesion Segmentation at 7 Tesla: Comparison of Single- and Multi-Contrast MRI Inputs

**DOI:** 10.3390/jimaging12100510

**Published:** 2026-10-14

**Authors:** Anna Petrova, Ivan Petrovic, Marc Berger, Yanniklas Kravutske, Joel Leinkauf, Rebeka Rumbak, Lukas Haider, Eva Niess, Bernhard Strasser, Wolfgang Bogner, Günther Grabner, Gabriel Bsteh, Thomas Berger, Paulus Rommer, Assunta Dal-Bianco, Stanislav Motyka

**Affiliations:** 1 High-Field MR Center, Department of Biomedical Imaging and Image-Guided Therapy, Medical University of Vienna, 1090 Vienna, Austria; 2 Department of Neurology, Medical University of Vienna, 1090 Vienna, Austria; 3 Comprehensive Center for Clinical Neurosciences & Mental Health, Medical University of Vienna, 1090 Vienna, Austria; 4 Division of Neuroradiology and Musculoskeletal Radiology, Department of Biomedical Imaging and Image-Guided Therapy, Medical University of Vienna, 1090 Vienna, Austria; 5 NMR Research Unit, Queen Square Multiple Sclerosis Centre, Queen Square Institute of Neurology, University College London, London WC1N 3BG, UK; 6 Comprehensive Center for AI in Medicine (CAIM), Medical University of Vienna, 1090 Vienna, Austria; 7 Christian Doppler Laboratory for MR Imaging Biomarkers (BIOMAK), Department of Biomedical Imaging and Image-Guided Therapy, Medical University of Vienna, 1090 Vienna, Austria; 8 Department of Medical Engineering, Carinthia University of Applied Sciences, 9020 Klagenfurt, Austria

**Keywords:** multiple sclerosis, MRI, lesion segmentation, deep learning, automated segmentation

## Abstract

Automated lesion segmentation can support rapid and reproducible quantitative assessment and long-term monitoring of multiple sclerosis. Ultra-high-field 7T MRI provides increased spatial resolution and lesion visibility but introduces challenges for automated analysis, including field inhomogeneity, susceptibility-related artifacts, and spatially varying image contrast. Although deep learning methods perform well at conventional field strengths, their application to 7T MRI remains underexplored. We evaluated nnUNet using FLAIR alone, MP2RAGE alone, and combined MP2RAGE+FLAIR. The dataset included 115 longitudinal 7T MRI scans from 57 patients with MS, with 20 scans from 11 independent patients reserved for testing. DeepMedic was included as a comparative architecture. On the test set, nnUNet achieved mean DSC values of 0.72, 0.71, and 0.66 for combined, MP2RAGE-only, and FLAIR-only inputs, respectively. No significant DSC differences were observed between nnUNet and DeepMedic. For both architectures, adding FLAIR to MP2RAGE did not show a statistically significant additional benefit in the present test cohort, whereas MP2RAGE-containing inputs significantly outperformed FLAIR-only input. The findings of the present study provide preliminary evidence suggesting that the observed voxel-level segmentation performance was primarily associated with the inclusion of MP2RAGE. However, lesion-level performance depended on lesion size, indicating further investigation of contrast-specific effects on small-lesion detection.

## 1. Introduction

Multiple sclerosis (MS) is a chronic autoimmune disease of the central nervous system affecting both white and gray matter. It is characterized by inflammation, demyelination, and axonal injury that ultimately leads to neurodegeneration and brain atrophy. As MS is most commonly diagnosed in young adults, with a higher prevalence in women [1], it carries a substantial long-term social and economic burden. Magnetic resonance imaging (MRI), in combination with clinical evaluation and cerebrospinal fluid (CSF) analysis, represents an important component of MS diagnosis [2]. In routine clinical practice, MRI at 1.5T and 3T field strengths provides adequate sensitivity for lesion detection and volumetric assessment [3,4]. However, ultra-high-field MRI at 7 Tesla (7T) offers increased spatial resolution and improved lesion visibility, enabling more detailed visualization of MS-specific imaging features such as the central vein sign (CVS) and paramagnetic rim lesions (PRLs), the latter representing a radiological marker of chronic active lesions [2,5]. These imaging features have recently been incorporated into the updated 2024 McDonald criteria to support diagnostic specificity in selected clinical scenarios [5].

Accurate detection and segmentation of MS lesions are essential for quantitative assessment of lesion burden and longitudinal disease activity. Lesion number, volume, location, and evolution over time provide relevant information on disease activity and treatment response. The distribution and temporal development of lesions may also support the assessment of dissemination in space and time [6]. Although manual segmentation is generally considered the reference standard, it is time-consuming, labor-intensive, and affected by intra- and inter-rater variability. These limitations reduce its scalability and reproducibility in large or longitudinal imaging datasets. Automated methods aim to address these limitations by providing faster and more consistent lesion measurements. However, automated segmentation remains challenging because of the heterogeneous appearance of MS lesions. Small, cortical, infratentorial, and confluent lesions may be particularly difficult to detect and delineate accurately. Additional challenges include the severe imbalance between lesion and non-lesion voxels, partial-volume effects, false-positive detections caused by vessels and imaging artefacts, and limited generalizability across scanners, acquisition protocols, and patient populations [7]. These factors are particularly relevant for deep learning methods, whose performance depends strongly on the characteristics of the training data.

At the same time, 7T MRI introduces several technical challenges that may affect automated segmentation performance. These include increased intensity inhomogeneity, spatially varying tissue contrast, susceptibility-related artifacts, differences in image appearance compared with conventional field strengths, and the need for accurate alignment between high-resolution image contrasts. The smaller voxel size also increases the computational requirements of three-dimensional segmentation and may increase the sensitivity of segmentation models to preprocessing and registration errors. Differences in imaging protocols, scanner characteristics, and magnetic field strength may therefore substantially influence lesion contrast and model generalizability [8].

Deep learning methods, particularly convolutional neural networks and U-Net architectures, have become widely used for automated MS lesion segmentation because they can learn complex spatial and intensity-based features directly from imaging data [7,9]. Most existing approaches, however, have been developed and evaluated on MRI acquired at conventional field strengths, typically 1.5T or 3T [9,10,11,12]. Their direct transfer to 7T MRI may therefore be limited by differences in spatial resolution, image contrast, signal characteristics, and artefact patterns.

Only a small number of studies have investigated MS lesion segmentation at 7T. La Rosa et al. [13] developed a deep learning framework for automated detection and classification of cortical MS lesions on 7T MRI. Donnay et al. [14] introduced a Pseudo-Label Assisted nnUNet approach for automated brain and lesion segmentation on 7T MRI using multiple contrasts derived from a single MP2RAGE acquisition. Maynord et al. [15] investigated UNETR- and SegFormer-based approaches for automated MS white matter lesion segmentation on 7T FLAIR MRI. Fartaria et al. [16] developed an automated method for the detection and segmentation of white matter and cortical MS lesions using MP2RAGE at 7T. However, differences across these studies in lesion targets, imaging contrasts, datasets, and segmentation methods make it difficult to determine the relative contribution of individual MRI contrasts to whole-brain MS lesion segmentation.

Magnetization-Prepared 2 Rapid Acquisition Gradient Echoes (MP2RAGE) and Fluid-Attenuated Inversion Recovery (FLAIR) contrasts provide complementary information for MS lesion assessment at 7T. MP2RAGE provides high-resolution T1-weighted anatomical contrast and is less sensitive to receive-field inhomogeneity than conventional T1-weighted acquisitions [17], while FLAIR provides complementary T2-weighted lesion contrast and is widely used for MS lesion detection. Compared with conventional field strengths such as 3T, FLAIR at 7T may be more affected by B0 and B1 field inhomogeneities and spatially varying contrast, which may influence lesion appearance and automated segmentation performance [18].

In particular, it remains unclear whether combining FLAIR and MP2RAGE provides a meaningful advantage over single-contrast input and whether the effect of contrast selection is consistent across different DL architectures. This comparison is also relevant for clinical translation. Multi-contrast approaches may provide complementary lesion information but require longer acquisition times, additional preprocessing, accurate inter-contrast registration, and the availability of all imaging sequences. In contrast, reliable segmentation using a single contrast could facilitate the analysis of incomplete or heterogeneous research datasets and simplify future implementation in clinical workflows.

The aim of this study was to evaluate single- and multi-contrast DL approaches for MS lesion segmentation using 7T MRI data. nnUNet [19] was used as the primary segmentation framework and evaluated using FLAIR and MP2RAGE, both individually and in combination. DeepMedic [20], trained and tested using the same patient-level data partitions, was included to assess whether the effect of contrast selection was consistent across two different DL architectures. The models were evaluated using both voxel-level and lesion-level metrics. Additionally, external automated segmentation methods were included as contextual reference methods without dataset-specific adaptation.

## 2. Materials and Methods

### 2.1. Patients

For the present analysis, we included 7T MRI scans acquired between 2023 and 2025 from 57 patients with MS participating in an ongoing longitudinal 7T MRI study at the Medical University of Vienna, initiated in 2010 [21].

All patients had clinically definite MS diagnosed at our Department of Neurology according to the revised McDonald criteria applicable at the respective time of diagnosis. Inclusion criteria at study enrolment were age > 18 years, EDSS ≤ 6.5, no corticosteroid treatment within the preceding 3 months, and no contraindications to 7T MRI. The study was approved by the local ethics committee (EC 154/2009), and all patients provided written informed consent in accordance with the Declaration of Helsinki [22].

The cohort consisted of 57 patients (32 female and 25 male), including 48 with relapsing-remitting MS (RRMS) and 9 with secondary-progressive MS (SPMS). Median age was 37.9 years (range, 22.9–75.7), median disease duration was 8.4 years (range, 0.08–45.2), and median EDSS was 1.0 (range, 0.0–7.5). Two patients had progressed beyond an EDSS of 6.5 after study enrolment. All patients underwent annual neurological examinations and 7T MRI scans, including FLAIR and MP2RAGE.

### 2.2. MR Imaging Protocol

All patients were scanned on a 7T whole-body MR scanner (Magnetom, Siemens Healthcare, Erlangen, Germany) with a 1TX/32RX head coil (Nova Medical, Wilmington, MA, USA). Two imaging contrasts were acquired during each session: (i) 3D T1-weighted MP2RAGE imaging (TR = 5000 ms, TE = 4.13 ms, TI1/TI2 = 700 ms/2700 ms, scan time = 8:02 min) at 0.8 mm × 0.8 mm × 0.8 mm resolution; (ii) 3D T2-weighted imaging using FLAIR (TR = 8000 ms, TE = 270 ms, TI = 2180 ms, scan time = 7:14 min) at 0.86 mm × 0.86 mm × 0.86 mm resolution.

### 2.3. Pre-Processing of MRI Data

First, FLAIR images were resampled to the spatial resolution of the MP2RAGE images using the mincresample tool (version 1.9.18.3) [23]. The resampled FLAIR images were then rigidly co-registered to the MP2RAGE images using FLIRT (version 5.5) from the FSL toolbox [24]. Subsequently, all images were corrected for intensity non-uniformity using the N4 bias field correction algorithm from the ANTs package (version 2.6.4) [25]. Following this, the intensity scales of the MR images were standardized by normalizing them to a range between 0 and 1. Normalization was performed using the following formula:

$$x_{norm}=\frac{x-x_{min}}{x_{max}-x_{min}}$$

where *x* is the original intensity value, and *x_min_* and *x_max_* are the minimum and maximum intensity values, respectively.

Brain extraction was performed using the Brain Extraction Tool from the FSL toolbox [24].

The same preprocessed images were used as input for all evaluated models to ensure comparability.

### 2.4. Training and Testing Data

We employed a longitudinal dataset consisting of 57 patients, resulting in a total of 115 scans. The sample size was fundamentally limited by the amount of suitable data within the existing 7T MRI cohort, and no formal a priori sample-size calculation was performed. Data splitting was performed at the patient level to prevent information leakage between the training and test datasets. All longitudinal scans from the same patient were assigned exclusively to a single data partition. In total, 95 scans from 46 patients were used for model training and validation, while 20 scans from 11 independent patients, including multiple longitudinal time points, were reserved as the test set. In total, the dataset contained 4664 lesions: 340 lesions of 0–5 mm^3^, 726 of 5–20 mm^3^, 1491 of 20–50 mm^3^, 1486 of 50–200 mm^3^, 350 of 200–500 mm^3^, 107 of 500–1000 mm^3^, and 164 lesions > 1000 mm^3^. The test set contained 917 lesions.

All lesions were manually segmented using ITK-SNAP (version 4.4.0) [26] on MP2RAGE and FLAIR images by three trained medical doctors (M.B., Y.K., and J.L.) experienced in MRI-based lesion segmentation in MS. The three raters annotated different subsets of the dataset rather than independently segmenting the same examinations. The segmentations were subsequently reviewed and refined by L.H., a senior radiologist with 10 years of experience in MRI in MS, and A.D.B., a neurologist with 15 years of experience in 7T MRI in MS. The reviewed and refined manual segmentations were used as the Ground truth (GT) for all experiments. For consistency, the same GT dataset was used for training, validation, and testing across all evaluated models. The lesion annotations in the test set were additionally reviewed by I.P. and confirmed by L.H.

### 2.5. Neural Network Architecture

In this study, two DL architectures, nnUNet (v2) [19] and DeepMedic (version 0.8.4) [20], were used for the automatic segmentation of lesions in MS.

The nnUNet model used the 3D full-resolution plain convolutional encoder configuration automatically generated by the nnUNet planning framework. The network consisted of six encoder–decoder stages with 32, 64, 128, 256, 320, and 320 feature maps, respectively. Each encoder stage contained two 3D convolutional layers, and each decoder stage contained two convolutional layers. All convolutional layers used 3 × 3 × 3 kernels. Spatial downsampling was performed using strides of 2 × 2 × 2 after the first stage, while skip connections transferred high-resolution spatial information from the encoder to the corresponding decoder stages. Instance normalization and LeakyReLU activation were used throughout the network, and no dropout was applied. The model operated on 128 × 128 × 128 voxel patches at an isotropic spacing of 0.75 mm. The remaining preprocessing and training parameters were automatically configured by the nnUNet framework.

DeepMedic was implemented as a multi-scale 3D CNN with parallel normal-resolution and subsampled pathways, enabling the network to combine local image information with broader spatial context. The normal-resolution pathway comprised 8 convolutional layers with 24, 24, 32, 32, 40, 40, 48, and 48 feature maps, whereas the subsampled pathway comprised 8 convolutional layers with 32, 32, 40, 40, 48, 48, 56, and 56 feature maps and used a subsampling factor of 3 × 3 × 3. The outputs of these pathways were subsequently combined and processed by a classification pathway containing two hidden layers with 128 and 64 feature maps before the final output layer. PReLU activations and batch normalization were used throughout the network. DeepMedic was included as a comparative deep learning baseline because it is an established multi-scale 3D CNN architecture that has been widely applied to brain lesion segmentation tasks [9]. A complete overview of the architectural characteristics of both models is provided in Supplementary Table S1.

### 2.6. Network Training

Each architecture was trained separately using three MRI input configurations to evaluate the contribution of each contrast to segmentation accuracy: (i) FLAIR only, (ii) MP2RAGE only, and (iii) combined MP2RAGE+FLAIR. All models were trained on an NVIDIA DGX workstation (NVIDIA Corporation, Santa Clara, CA, USA) using a single NVIDIA Tesla V100 GPU with 32 GB of memory. Both architectures were trained and evaluated using the same patient-level data partitions and the same preprocessed images to ensure a consistent comparison.

The nnUNet v2 model was trained for 1000 epochs using five-fold cross-validation. Training of each fold required approximately 19 h, corresponding to approximately 95 h for all five folds per input configuration. The network architecture was automatically selected by the nnUNet framework and remained unchanged during training.

The DeepMedic model was trained for 80 epochs, with a total training time of approximately 24 h. The network configuration was based on the original authors’ recommendations, with manual adjustment of selected hyperparameters. The training segment size was set to 45 × 45 × 45 voxels, the batch size to 3 and the number of sub-epochs was reduced to 10 per epoch. Optimization was performed using the Adam optimizer with an initial learning rate of 2 × 10^−4^. A weighted sum of Cross-entropy (relative weight 0.3) and Dice (relative weight 0.7) loss was used for training. The same DeepMedic hyperparameter configuration was applied to all three input configurations. A complete list of hyperparameters and architectural features of both models is provided in Supplementary Table S1.

Inference time was additionally measured for both nnUNet v2 and DeepMedic on the test dataset using the same computational environment. The average processing time was 87.5 s per scan for nnUNet v2 and 30.0 s per scan for DeepMedic. GPU memory usage was monitored during inference using nvidia-smi and the maximum observed process-level memory usage was recorded. Additional computational characteristics, including the number of trainable parameters, FLOPs, and peak observed GPU memory usage, are provided in Supplementary Table S2.

### 2.7. Evaluation and Comparison

Segmentation performance was evaluated at both the voxel and lesion levels. At the voxel level, several metrics were calculated to assess agreement between the predicted and ground-truth (GT) masks. These included the Dice similarity coefficient (DSC), Intersection over Union (IoU), Matthew’s correlation coefficient (MCC), sensitivity, positive predictive value (PPV), false discovery rate, and false negative rate, as defined in Supplementary Table S3.

At the lesion level, each labelled lesion in the GT was compared with the corresponding predicted lesion using connected-component analysis. Each GT lesion was considered detected when the overlap with at least one predicted component reached the predefined threshold. Lesions were classified as true positive, false positive, or false negative according to the voxel overlap between the GT and predicted masks. Two overlap thresholds were applied: (i) at least 1% of the GT lesion volume, representing a minimal criterion for lesion detection, and (ii) at least 30% of the GT lesion volume, representing a more substantial overlap between the GT and prediction.

Model performance was additionally compared with two external baseline methods: the publicly available deep learning method MindGlide (version 1.0.1) [27] and the non-deep-learning Lesion Growth Algorithm (LGA) (version 3.0.0) [28].

MindGlide, proposed by Goebl et al. [27], employs a 3D CNN architecture designed for brain and lesion segmentation in MRI scans of patients with MS. The model was trained on 4247 in vivo scans from 2871 patients acquired using 592 MRI scanners at field strengths of 1.5T and 3T. Multiple MRI contrasts, including FLAIR and T1-weighted images, were used during training. In the present study, MindGlide was applied to all 20 test scans using either FLAIR or MP2RAGE as input.

LGA [28] combines FLAIR and T1-weighted images to generate lesion segmentations; in the present study, MP2RAGE was provided as the T1-weighted input. It initializes lesion probability maps using tissue probability maps and FLAIR hyperintensities and subsequently refines the segmentation based on intensity and spatial information. LGA was applied using its default parameters. Its segmentation outputs were processed and evaluated using the same procedures and metrics as the DL-based methods.

MindGlide and LGA were included as reference methods to provide contextual baseline comparisons, albeit without extensive dataset-specific fine-tuning or retraining. Thus, the reported performance of these baseline methods may be further improved through thorough adaptation to this specific application.

### 2.8. Statistical Analysis

Paired comparisons of voxel-level DSC were performed at the patient level using two-sided Wilcoxon signed-rank tests. For patients with multiple longitudinal scans, DSC values were first averaged across scans so that each patient contributed one value to the statistical analysis. To account for multiple comparisons, *p*-values were adjusted using the Benjamini–Hochberg false discovery rate procedure within each predefined family of comparisons. Effect sizes were reported as rank-biserial correlations. Using patient-level cluster bootstrap resampling with 10,000 repetitions, 95% confidence intervals for mean DSC were estimated, preserving all longitudinal scans belonging to each resampled patient. An adjusted *p*-value < 0.05 was considered statistically significant.

To assess the association between lesion size and lesion detection while accounting for clustering of multiple lesions within the same patient, generalized estimating equation (GEE) logistic regression models with a binomial distribution were fitted separately for nnUNet v2 and DeepMedic using the combined MP2RAGE+FLAIR input. For the lesion-size analysis, a lesion was considered detected if at least one predicted voxel overlapped the ground-truth lesion. Lesion detection was used as the binary outcome and the natural logarithm of lesion volume as the predictor. In addition, detection of lesions measuring 5–20 mm^3^ was directly compared with that of lesions measuring 50–200 mm^3^ using GEE logistic regression for each model and input configuration, with patient ID as the clustering variable.

To further characterize the statistical sensitivity of the patient-level comparisons, two complementary power analyses were performed. For each paired comparison, descriptive post hoc achieved power was calculated based on the observed standardized paired effect size *d_z_*, defined as:

$$d_{z}=\frac{\bar{D}}{s_{D}}$$

where $\bar{D}$ is a mean paired difference and *s_D_* is the standard deviation of the paired difference.

Statistical power was estimated using Monte Carlo simulation with 200,000 repetitions for a two-sided Wilcoxon signed-rank test at α = 0.05, assuming normally distributed standardized paired differences under a location-shift model. Because observed post hoc power is closely related to the observed effect size and *p*-value, these estimates were considered descriptive. A power sensitivity analysis was also performed to determine the minimum standardized paired effect that could be detected with 80% power given the available sample of 11 independent patients. As an additional sensitivity analysis, the multiple-comparison correction was repeated using the Holm procedure within the same predefined families of comparisons.

**Figure 1:**
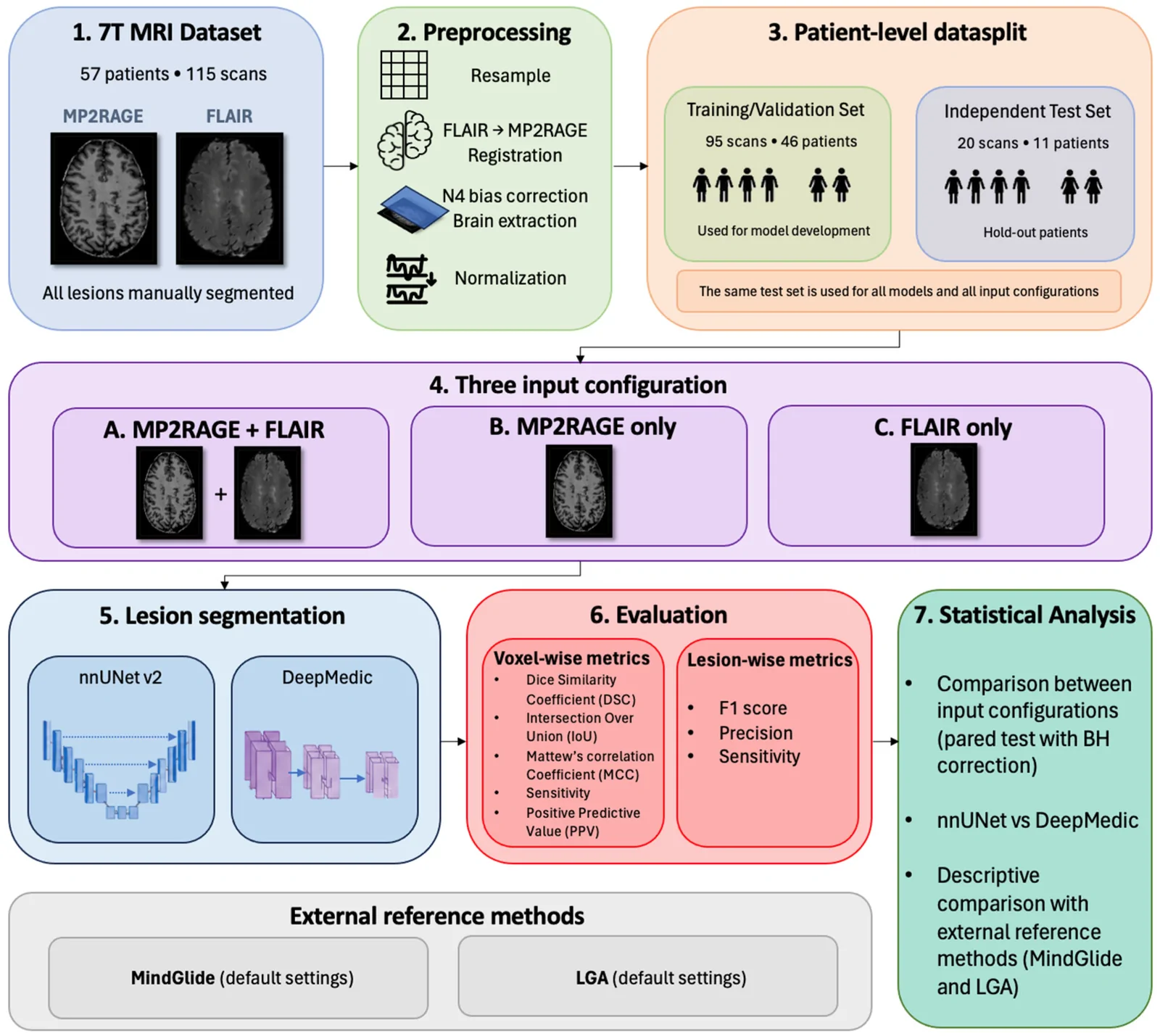
Overview of the study workflow. The pipeline includes 7T MRI preprocessing, patient-level dataset splitting, evaluation of three MRI input configurations (MP2RAGE+FLAIR, MP2RAGE-only, and FLAIR-only), lesion segmentation using nnUNet v2 and DeepMedic, and subsequent voxel- and lesion-level performance evaluation.

Given the small number of independent patients available for testing (*n* = 11), the statistical results should be considered preliminary and interpreted with caution. Adjusted *p*-values were reported alongside effect sizes, confidence intervals, and descriptive performance measures. An overview of the complete study workflow is provided in Figure 1.

## 3. Results

### 3.1. Voxel-Level Segmentation Performance

**Figure 2:**
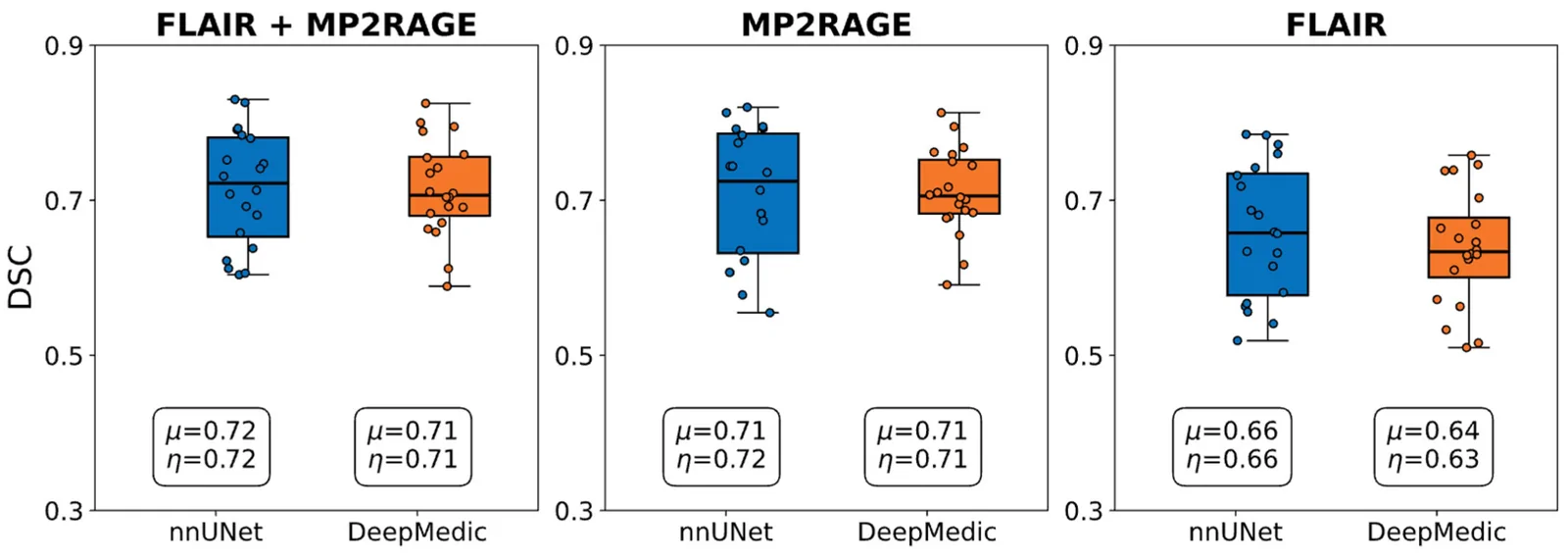
Voxel-level DSC distributions for nnUNet and DeepMedic across the three input configurations. Each point represents the DSC of a single test case; *μ* indicates the mean and *η* indicates the median.

The nnUNet model achieved a mean DSC of 0.72 for combined MP2RAGE+FLAIR input, 0.71 for MP2RAGE alone, and 0.66 for FLAIR alone (Table 1, Figure 2). DeepMedic achieved corresponding mean DSC values of 0.71, 0.71, and 0.64, respectively. Patient-level paired Wilcoxon signed-rank tests did not show statistically significant differences in DSC between nnUNet and DeepMedic for any of the three input configurations after correction for multiple comparisons (adjusted *p* = 0.638 for MP2RAGE+FLAIR, adjusted *p* = 0.638 for MP2RAGE, and adjusted *p* = 0.369 for FLAIR).

Across the evaluated input configurations, nnUNet generally showed higher PPV and similar or slightly higher IoU and MCC values compared with DeepMedic, whereas DeepMedic demonstrated higher sensitivity. Secondary voxel-level metrics are reported in Table 1 and Supplementary Table S4. 

**Figure 3:**
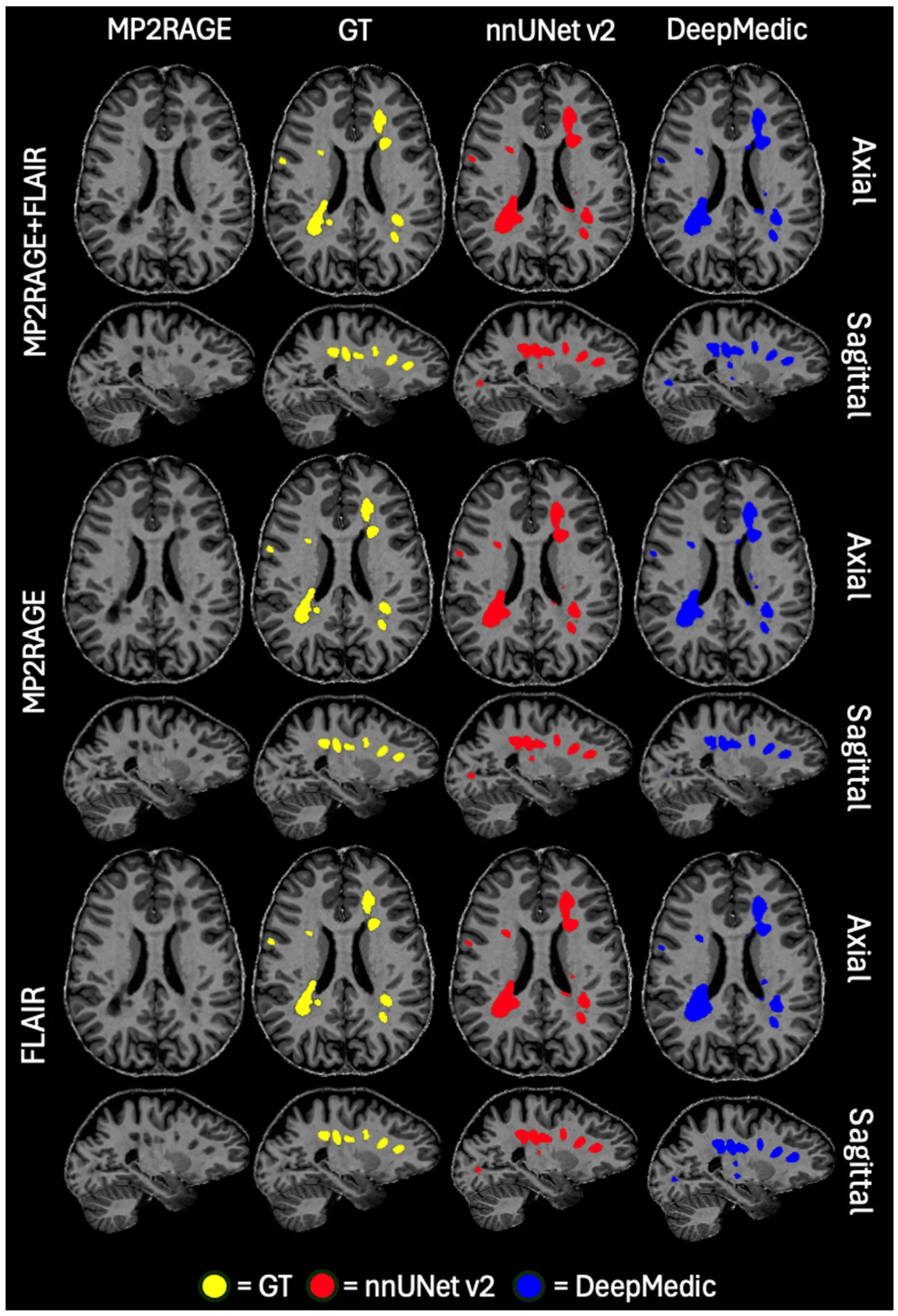
Representative segmentation results for nnUNet and DeepMedic across different MP2RAGE and FLAIR input configurations in one test case. Ground truth lesions are shown in yellow, nnUNet predictions in red, and DeepMedic predictions in blue. Representative slices are shown for qualitative illustration of segmentation performance.

Within nnUNet, combined MP2RAGE+FLAIR input did not significantly outperform MP2RAGE alone (adjusted *p* = 0.175; rank-biserial r = 0.485). In contrast, both combined MP2RAGE+FLAIR and MP2RAGE-only input achieved significantly higher DSC than FLAIR-only input (adjusted *p* = 0.003 and adjusted *p* = 0.015, respectively). A similar pattern was observed for DeepMedic: combined input did not significantly differ from MP2RAGE alone (adjusted *p* = 0.240; rank-biserial r = 0.424), whereas both MP2RAGE-containing configurations significantly outperformed FLAIR-only input (both adjusted *p* = 0.001). Representative segmentation results for nnUNet and DeepMedic across the three MRI input configurations are shown in Figure 3.

Descriptive post hoc achieved power varied according to the magnitude of the observed paired effects and ranged from 4.4% to 99.7% across comparisons. The power sensitivity analysis showed that, with 11 independent paired observations, a standardized paired effect of approximately |*d_z_*|= 0.99 was required to achieve 80% power using a two-sided Wilcoxon signed-rank test at α = 0.05.

The available test cohort was primarily sensitive to large paired effects, whereas smaller or moderate differences may not be reliably detected. The pattern of statistically significant and non-significant comparisons remained unchanged when the Holm correction was applied instead of the Benjamini–Hochberg procedure.

Secondary metrics, including FDR and FNR, are reported in Supplementary Table S4. Full pairwise statistical comparisons, including raw, Benjamini–Hochberg-adjusted and Holm-adjusted *p*-values, rank-biserial correlations, standardized paired effect sizes *d_z_*, and descriptive post hoc power estimates, are provided in Supplementary Tables S5–S7. 

### 3.2. Lesion-Level Detection Performance

**Figure 4:**
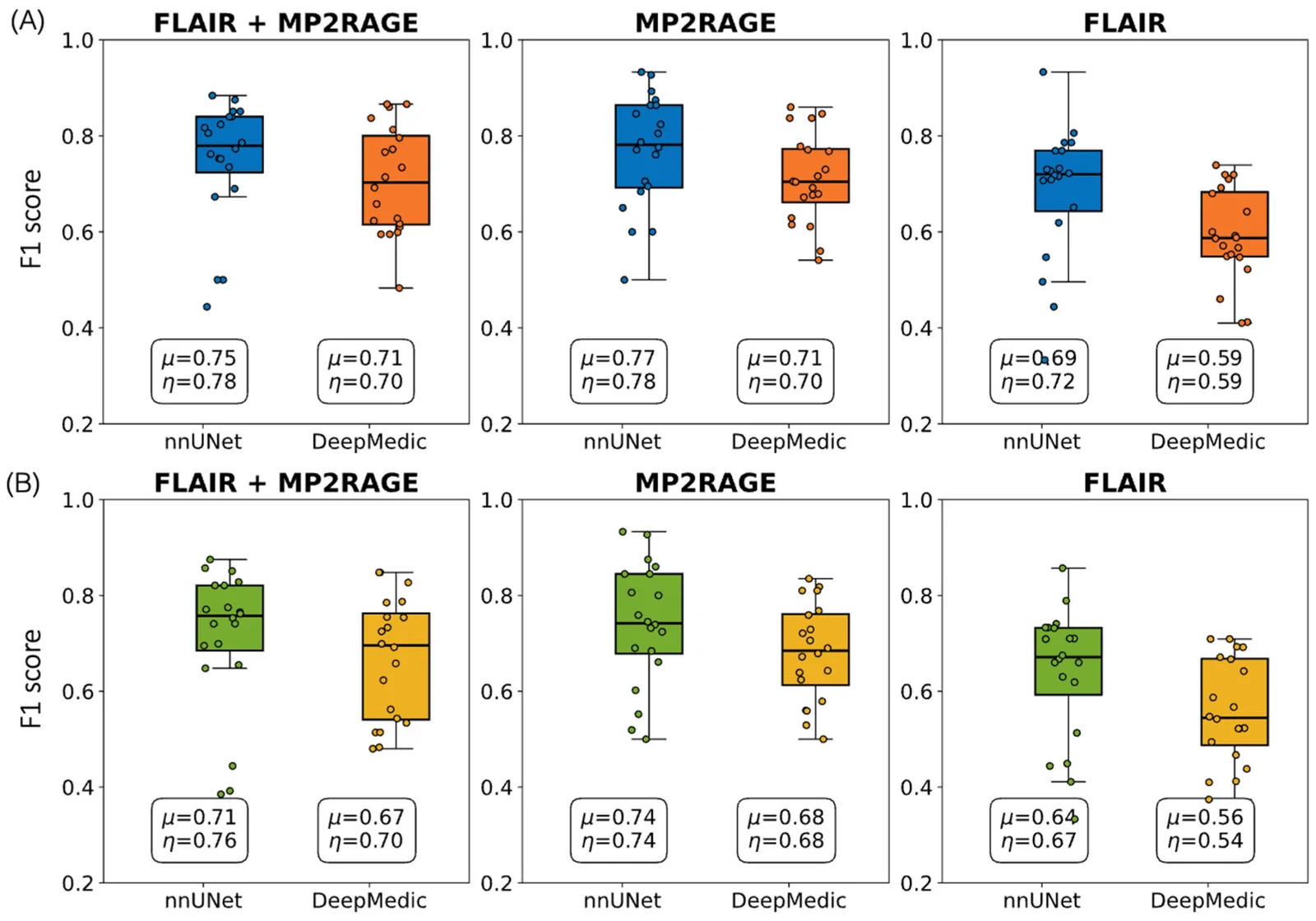
Lesion-level F1 score distributions for nnUNet and DeepMedic at two overlap thresholds: (**A**) 1% and (**B**) 30%. Each point represents the F1 score of a single test case; *μ* indicates the mean and *η* indicates the median. (In panel (**A**), blue and orange represent nnUNet and DeepMedic, respectively; in panel (**B**), green and yellow represent nnUNet and DeepMedic, respectively.)

At the lesion level, nnUNet showed comparable mean performance with combined MP2RAGE+FLAIR and MP2RAGE-only input, with MP2RAGE-only achieving the highest mean F1 values at both overlap thresholds (Table 2, Figure 4). At the 1% overlap threshold, nnUNet achieved F1 values of 0.75 for combined MP2RAGE+FLAIR, 0.77 for MP2RAGE alone, and 0.69 for FLAIR alone. Corresponding PPVs were 0.84, 0.86, and 0.85. At the 30% threshold, F1 values were 0.71, 0.74, and 0.64, respectively.

For DeepMedic with combined MP2RAGE+FLAIR input, F1 and PPV were 0.71 and 0.64 at the 1% threshold and 0.67 and 0.62 at the 30% threshold, respectively. Across input configurations, DeepMedic generally showed higher sensitivity but lower PPV than nnUNet, consistent with a higher proportion of false-positive detections.

All metrics for 20 test cases for nnUNet and DeepMedic are reported in Supplementary Tables S8 and S9.

### 3.3. Additional Comparisons with External Reference Methods

**Figure 5:**
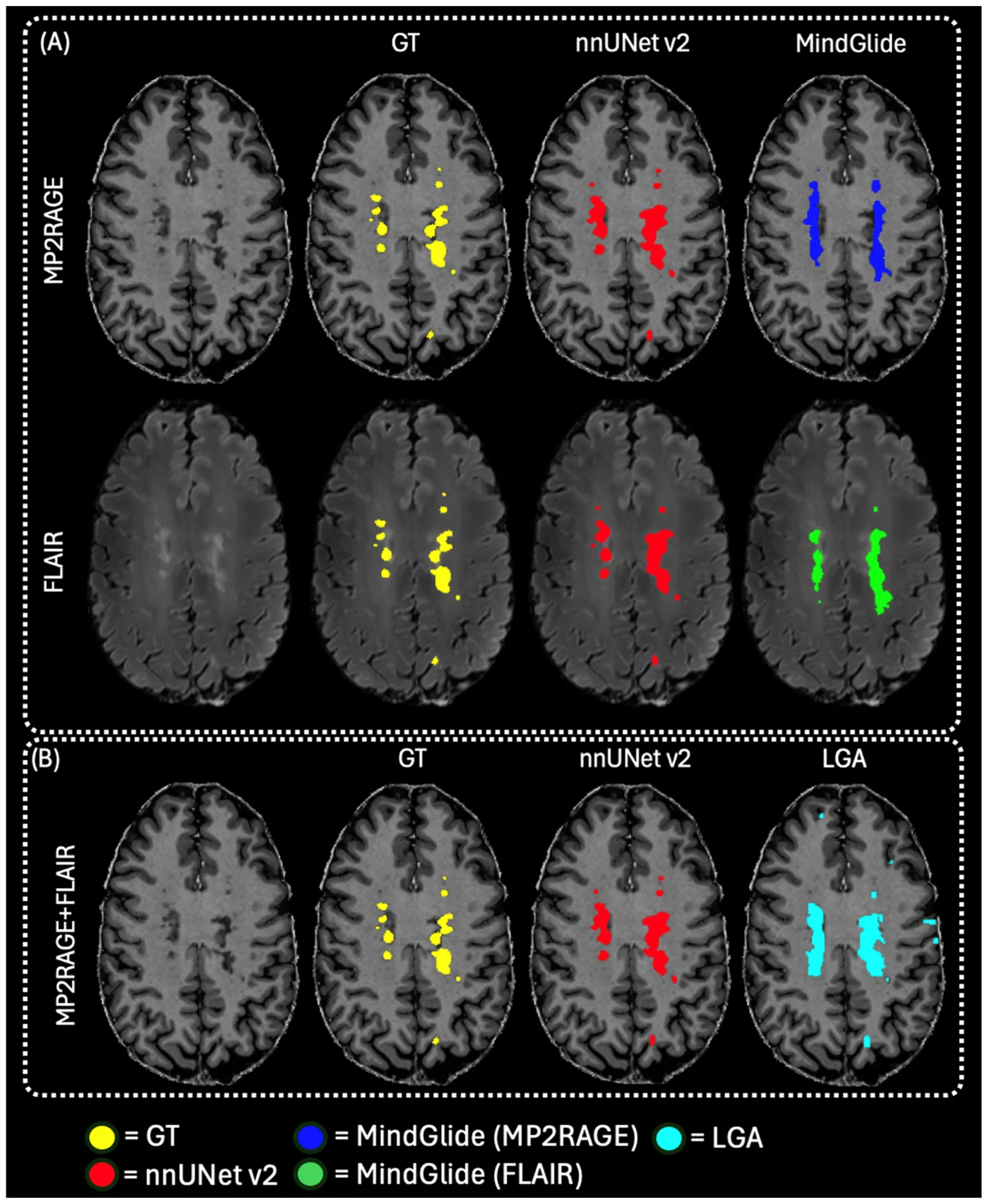
Representative comparison of nnUNet with reference automated segmentation methods. (**A**) Single-contrast comparison of nnUNet and MindGlide. Ground truth lesions are shown in yellow, nnUNet predictions in red, and MindGlide predictions in blue/green. (**B**) Combined MP2RAGE+FLAIR comparison of nnUNet and LGA. Ground truth lesions are shown in yellow, nnUNet predictions in red, and LGA predictions in light blue. Representative slices are shown for qualitative illustration of segmentation performance.

In the present test set, comparisons with the external reference methods showed higher observed performance values for nnUNet for the corresponding input configurations (Table 3, Figure 5). MindGlide achieved a mean DSC of 0.38 using MP2RAGE-only input, whereas nnUNet achieved a mean DSC of 0.71 using the same input contrast. LGA achieved a mean DSC of 0.33 using the combined MP2RAGE+FLAIR input. The corresponding voxel-level performance metrics are reported in Supplementary Table S10.

### 3.4. Lesion Detection Performance by Lesion Volume

**Figure 6:**
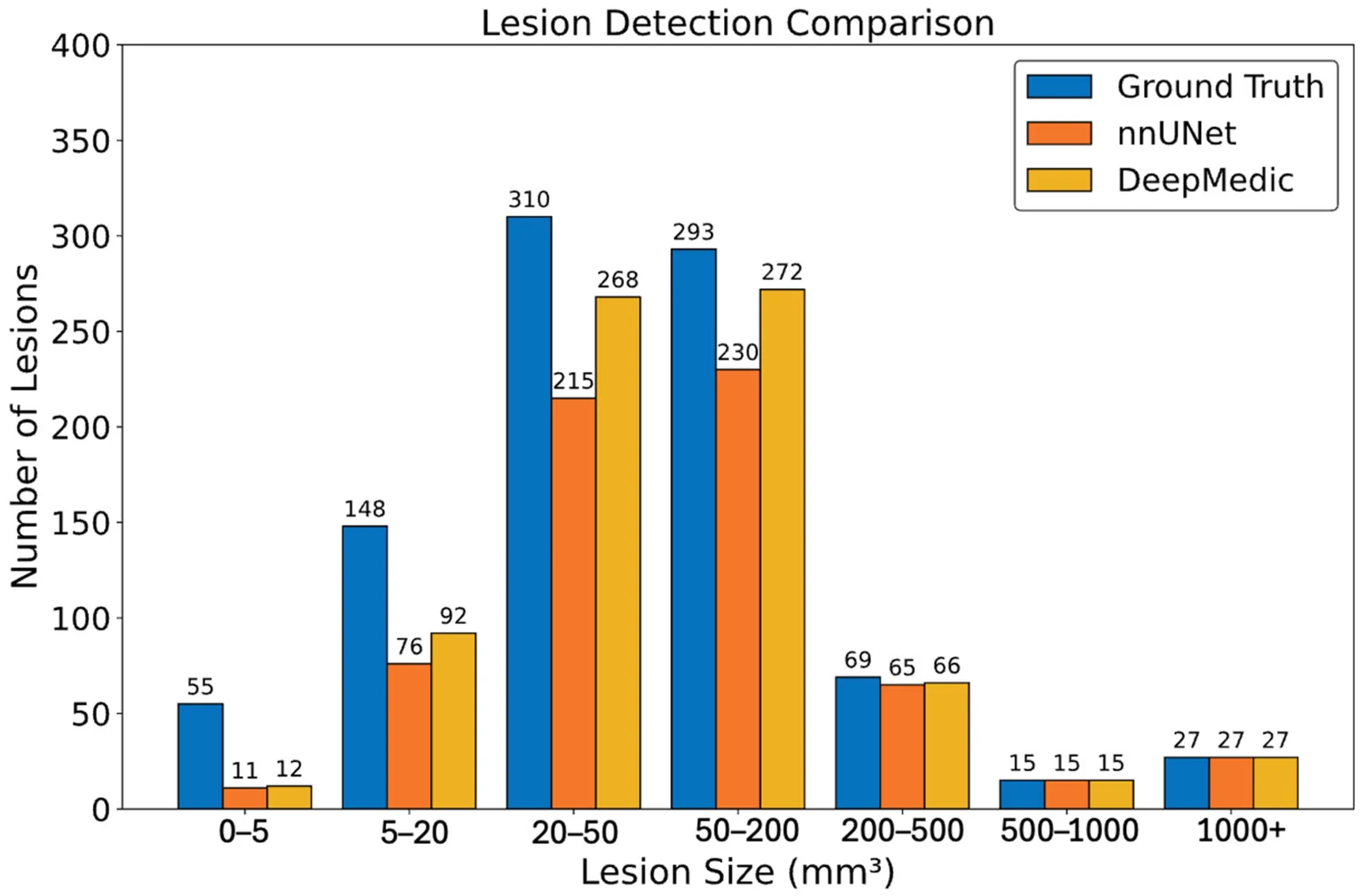
Number of ground-truth lesions and lesions detected by nnUNet and DeepMedic across lesion-volume categories using combined MP2RAGE+FLAIR input. For this lesion-size analysis, a lesion was considered detected if at least one predicted voxel overlapped the ground-truth lesion.

Lesion detection performance increased with lesion size for both architectures (Figure 6). This association was confirmed by GEE logistic regression using log-transformed lesion volume, with increasing lesion volume associated with higher odds of detection for both nnUNet v2 (OR = 1.92, 95% CI: 1.72–2.14, *p* < 0.001) and DeepMedic (OR = 2.33, 95% CI: 1.97–2.74, *p* < 0.001) using combined MP2RAGE+FLAIR input. Specifically, lesions measuring 50–200 mm^3^ were detected significantly more frequently than lesions measuring 5–20 mm^3^ (nnUNet v2: 78.5% vs. 51.4%, OR = 3.46, 95% CI: 2.68–4.46, *p* < 0.001; DeepMedic: 92.8% vs. 62.2%, OR = 7.88, 95% CI: 4.47–13.89, *p* < 0.001). These findings indicate a clear dependence of lesion detection performance on lesion size. Lesion detection results across lesion-volume categories for models using MP2RAGE-only or FLAIR-only inputs are provided in Supplementary Figure S1. The results of the GEE logistic regression directly comparing detection of lesions measuring 5–20 mm^3^ with lesions measuring 50–200 mm^3^ across all model and input configurations are provided in Supplementary Table S11.

### 3.5. Verification of False Positive Segmentations

**Figure 7:**
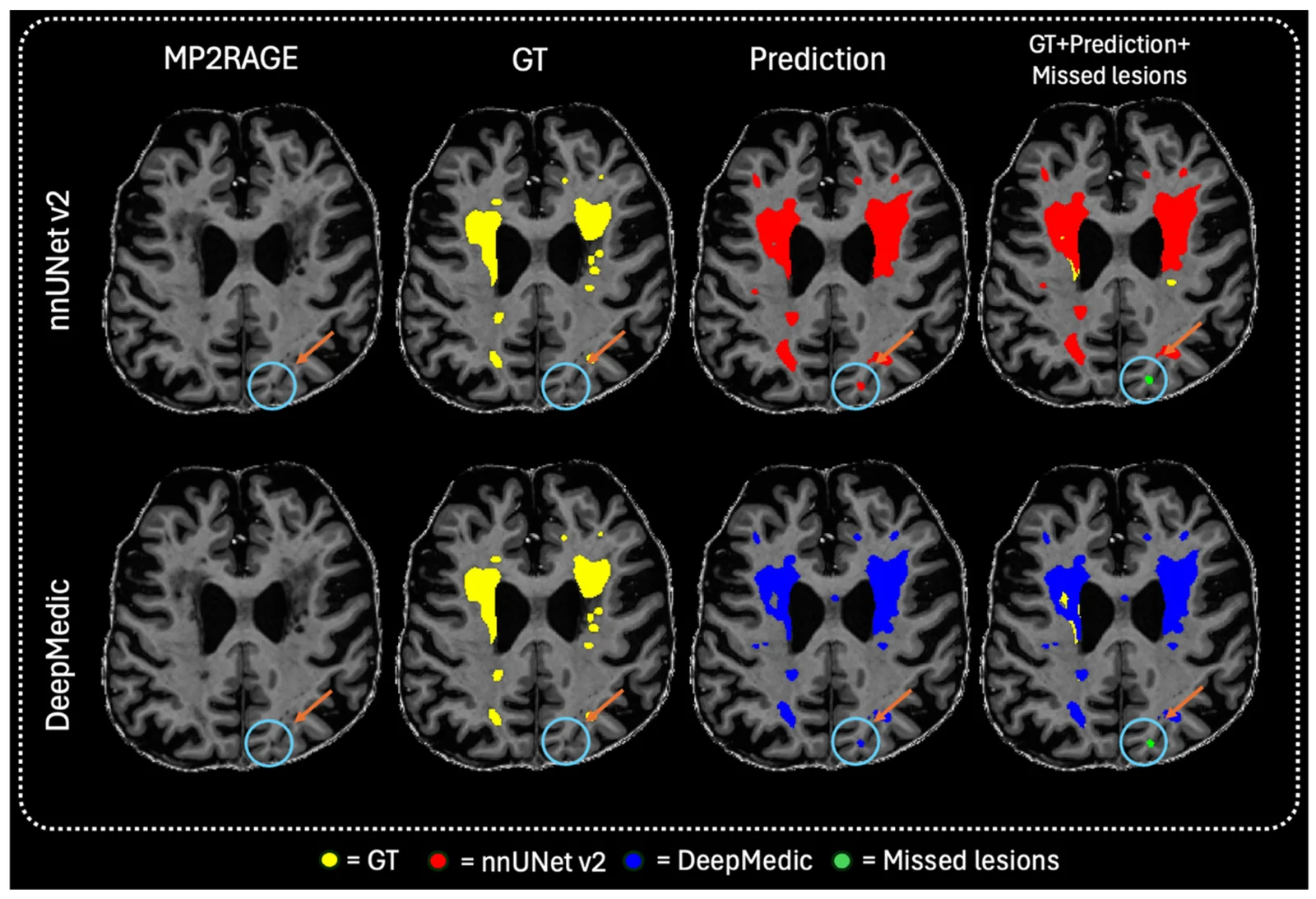
Examples of lesions missed during manual segmentation but detected by the deep learning models. Yellow indicates the manual ground-truth mask, red indicates nnUNet predictions, blue indicates DeepMedic predictions, and green indicates lesions identified during expert reassessment as missed in the original manual segmentation. (Orange arrows and light blue circles highlight lesions missed in the original manual segmentation but detected by the deep learning models).

The models also detected lesions that had been missed during the original manual segmentation (Figure 7). To further assess this, apparent false-positive detections generated by nnUNet and DeepMedic using the combined MP2RAGE+FLAIR input were independently reviewed by three experienced raters. The re-evaluation showed that 63% of apparent false-positive nnUNet detections and 60% of apparent false-positive DeepMedic detections for the combined MP2RAGE+FLAIR input corresponded to true lesions on expert reassessment. Most of the remaining false-positive detections corresponded to vascular structures or were located in cortical and infratentorial regions, where lesion detection is particularly challenging because of field inhomogeneities, anatomical complexity, and reduced local lesion-to-background contrast.

## 4. Discussion

This study evaluated automated lesion segmentation in MS using DL approaches on in-house 7T multi-contrast MRI data. The nnUNet architecture was employed as the primary segmentation model and trained using different contrast combinations, including single-contrast inputs (MP2RAGE or FLAIR) and a dual-contrast combination (MP2RAGE+FLAIR), to assess their impact on segmentation accuracy. Baseline comparisons were performed against DeepMedic, MindGlide, and LGA. DeepMedic was trained on the same data as the nnUNet model, whereas MindGlide and LGA were included as additional contextual baseline methods without application-specific optimization. Our results indicate that nnUNet achieved its highest voxel-level DSC with combined MP2RAGE+FLAIR input, although performance was not significantly different from MP2RAGE alone. No statistically significant DSC differences were observed between nnUNet and DeepMedic for any input configuration. Importantly, both architectures showed the same contrast-related pattern: MP2RAGE-containing inputs significantly outperformed FLAIR-only input, whereas adding FLAIR to MP2RAGE did not result in a statistically significant additional benefit.

The small number of independent patients in the test set (*n* = 11) limits the statistical sensitivity of the present study. The power sensitivity analysis showed that a standardized paired effect of approximately |*d_z_*| = 0.99 was required to achieve 80% power at α = 0.05, indicating that the available test cohort was primarily sensitive to large paired effects. Consequently, small or moderate differences between models or input configurations may have remained undetected, and non-significant comparisons should not be interpreted as evidence of equivalence. This is particularly relevant to comparisons between MP2RAGE+FLAIR and MP2RAGE-only, for which the observed effects were smaller. In contrast, comparisons involving FLAIR-only input showed larger observed effects. Nevertheless, the statistical findings should be interpreted together with effect sizes, confidence intervals, and descriptive performance measures.

In recent years, approval by the U.S. Food and Drug Administration has contributed to the gradual introduction of 7T ultra-high-field MRI into clinical practice, including applications in neurological imaging [29]. The increasing availability of 7T MRI creates a need for automated methods capable of processing high-resolution images in a reproducible manner. Reliable lesion segmentation could facilitate quantitative assessment of lesion burden and longitudinal lesion evolution in research settings and, following further validation, may support future clinical workflows.

Segmentation performance was evaluated at both the voxel and lesion levels, providing complementary information on spatial overlap and lesion detection. Voxel-level metrics may be dominated by larger lesions because of the greater number of voxels they contain. Despite this, many published studies report only DSC, which, as noted by Reinke et al. [30], does not fully characterize segmentation performance, since DSC may remain relatively high despite missed small lesions, particularly when performance is dominated by larger lesion volumes, and provides limited information about false-positive lesion detections. Some studies additionally report lesion-level measures, such as lesion true-positive and false-positive ratios, which provide complementary information but do not fully characterize lesion localization and overlap quality. These limitations significantly impact the evaluation of MS lesion segmentation quality and are critical factors for understanding model performance and its potential clinical applicability.

The clinical significance of small differences in segmentation metrics, such as an absolute difference of 0.01 in DSC, was not assessed in the present study. The primary aim was to compare segmentation performance across MRI input configurations rather than to establish associations between segmentation accuracy and clinical outcomes. Future studies should therefore investigate whether differences in automated segmentation performance translate into clinically meaningful differences in longitudinal lesion burden, disease activity, EDSS progression, or other measures of clinical outcome.

MindGlide and LGA were included as additional deep learning and conventional baselines to provide context for the performance observed in the present study. These methods were, however, not retrained or fine-tuned for this specific application. The lower performance observed for MindGlide in the present dataset might therefore reflect a mismatch between its training domain, which included large 1.5T and 3T MRI datasets, and the 7T data used in this study. In addition, differences in network architecture, training data, preprocessing, and target definitions may have contributed to the observed performance differences. Similarly, LGA was evaluated using its default parameters without dataset-specific optimization. Thus, the baseline methods may achieve improved performance when optimized for the present application, and the reported results do not permit conclusions regarding the superior achievable performance of nnUNet on 7T MRI data. These comparisons were included to provide contextual reference points for automated multiple sclerosis lesion segmentation within the present dataset.

Regarding the effect of input contrast, several factors may have contributed to the better segmentation performance of MP2RAGE-containing configurations compared with FLAIR-only input. MP2RAGE provides high-resolution anatomical information and relatively homogeneous T1-weighted contrast, which may facilitate lesion delineation at 7T [17]. In contrast, FLAIR at ultra-high field may be more affected by spatially varying signal intensity and field inhomogeneities, potentially reducing the consistency of lesion appearance across the brain [18]. These characteristics may partly explain the better performance of MP2RAGE-containing input configurations observed in the present dataset. However, the current study was not designed to determine the specific imaging mechanisms underlying these differences.

Beyond the overall segmentation performance, lesion size was an important factor influencing lesion-level detection, particularly because very small lesions were included in the primary analysis. To assess the effect of lesion size on segmentation performance, an additional sensitivity analysis was performed after excluding lesions smaller than 3 mm^3^ and 10 mm^3^ [6,31]. The results of this sensitivity analysis are provided in Supplementary Figures S2–S4.

Consistent with the sensitivity analysis, lesion detection increased significantly with lesion volume for both architectures using combined MP2RAGE+FLAIR input (GEE logistic regression using log-transformed lesion volume, both *p* < 0.001). In the direct comparison of lesion-size categories, lesions measuring 50–200 mm^3^ had significantly higher odds of detection than lesions measuring 5–20 mm^3^ across all configurations (all *p* < 0.001). The odds ratios ranged from 3.46 (95% CI: 2.68–4.46) for nnUNet v2 using combined MP2RAGE+FLAIR input to 9.95 (95% CI: 6.21–15.92) for DeepMedic using FLAIR-only input.

The reduced detection performance observed for small lesions also highlights an important limitation related to the manually defined Ground truth. Despite the high level of expertise of the specialists and additional expert review, manual lesion segmentation remains subjective and may result in small lesions being overlooked. To investigate this issue, apparent false-positive detections generated by nnUNet and DeepMedic using the combined MP2RAGE+FLAIR input in the test dataset were independently reassessed by three additional experts. The reassessment confirmed that 63% of apparent false-positive nnUNet detections and 60% of apparent false-positive DeepMedic detections corresponded to lesions that had been missed in the original manual GT. These findings suggest that the models were able to identify some lesions missed during manual segmentation and also highlight the difficulty of defining reliable Ground truth for complex tasks such as MS lesion segmentation. Similar annotation omissions may also be present in the training data, although this was not systematically assessed in the present study. Furthermore, because the primary raters annotated different subsets of the dataset rather than independently segmenting the same cases, formal inter-rater agreement could not be quantified, which represents an additional limitation of the present study. Additional results from the reassessment of apparent false-positive detections for the single-contrast configurations are provided in Supplementary Table S12.

The performance of the combined MP2RAGE+FLAIR configuration may also have been influenced by the alignment between the two input contrasts. FLAIR images were rigidly registered to the corresponding MP2RAGE images while preserving the anatomical geometry of the acquired images. Nevertheless, rigid registration may not fully compensate for local inter-contrast misalignment, particularly in regions affected by susceptibility-related distortions and field inhomogeneities at 7T. Residual misregistration may therefore have affected multi-contrast segmentation performance, particularly for small lesions, and should be considered a limitation of the present study.

Another consideration is the difference in model configuration strategies. nnUNet automatically adapts its architecture and training configuration to the characteristics of the dataset, whereas the DeepMedic configuration was manually specified based on the recommendations of the original framework. Consequently, the degree of optimization was not identical between the two architectures, and additional hyperparameter tuning of DeepMedic could potentially affect its performance. The comparison between nnUNet and DeepMedic should therefore be interpreted as a comparison of the implemented configurations rather than an exhaustive comparison of the maximum achievable performance of the two architectures.

In addition, the relatively limited number of independent subjects represents an important limitation of this study. Although the dataset comprised 115 longitudinal MRI examinations from 57 patients, the number of independent subjects remains relatively limited for training and evaluating deep learning models. To prevent information leakage between longitudinal examinations, data splitting was performed at the patient level, ensuring that all scans from the same patient were assigned to the same subset. Nevertheless, the limited cohort size and absence of external validation may affect the generalizability of the findings. Future studies should therefore validate these results in larger, independent, and preferably multicenter 7T cohorts. Furthermore, because all data were acquired at 7T using a single scanner and imaging protocol, the present findings should be regarded as preliminary and specific to the evaluated single-center 7T dataset and may not directly generalize to conventional 1.5T or 3T clinical MRI or to 7T data acquired using different scanners and acquisition protocols. Future work should also investigate attention-based architectures, including approaches incorporating the Convolutional Block Attention Module (CBAM), as a potential strategy for improving segmentation performance, particularly for small lesions [32,33].

## 5. Conclusions

This study demonstrates the feasibility of deep learning-based MS lesion segmentation using high-resolution 7T MRI. MP2RAGE-containing input configurations achieved significantly better voxel-level segmentation performance than FLAIR-only input, whereas no statistically significant improvement in DSC from adding FLAIR to MP2RAGE was detected in the present test cohort. However, lesion-level performance was strongly dependent on lesion size, and the relative contribution of individual MRI contrasts to the detection and segmentation of small lesions requires further investigation. The present findings suggest that the observed voxel-level segmentation performance may have been primarily associated with the inclusion of MP2RAGE, while no statistically significant additional benefit of FLAIR was detected in the present test cohort. Given the limited number of independent patients in the test set and the use of a single scanner and acquisition protocol, the present findings require validation in larger independent and multicenter 7T cohorts. Overall, these findings highlight the potential of high-resolution 7T MRI combined with deep learning for automated quantitative assessment of MS lesion burden.

## Figures and Tables

**Table 1:** Voxel-level segmentation performance of nnUNet and DeepMedic across the three MRI input configurations.

Model	Input	DSC ± SD	95% CI	IoU	Sensitivity	PPV	MCC
nnUNet	MP2RAGE+FLAIR	0.72 ± 0.07	0.69–0.75	0.56	0.69	0.79	0.73
	MP2RAGE	0.71 ± 0.08	0.67–0.74	0.55	0.68	0.78	0.72
	FLAIR	0.66 ± 0.09	0.62–0.70	0.50	0.59	0.79	0.67
DeepMedic	MP2RAGE+FLAIR	0.71 ± 0.06	0.70–0.73	0.56	0.76	0.70	0.72
	MP2RAGE	0.71 ± 0.06	0.69–0.73	0.55	0.75	0.70	0.72
	FLAIR	0.64 ± 0.07	0.61–0.67	0.47	0.70	0.63	0.65

**Table 2:** Lesion-level detection performance of nnUNet and DeepMedic at 1% and 30% overlap thresholds.

Model	Input	Threshold 1%			Threshold 30%		
		Sens	PPV	F1	Sens	PPV	F1
nnUNet	MP2RAGE+FLAIR	0.71	0.84	0.75	0.66	0.83	0.71
	MP2RAGE	0.71	0.87	0.77	0.67	0.86	0.74
	FLAIR	0.61	0.85	0.69	0.55	0.83	0.64
DeepMedic	MP2RAGE+FLAIR	0.82	0.64	0.71	0.75	0.62	0.67
	MP2RAGE	0.81	0.65	0.71	0.76	0.63	0.68
	FLAIR	0.72	0.53	0.59	0.67	0.51	0.56

Values represent the mean of lesion-level metrics calculated separately for each of the 20 test scans.

**Table 3:** Descriptive comparison of nnUNet with external reference automated segmentation methods.

Model	Type	Input	DSC ± SD	Sensitivity	PPV	MCC
nnUNet	DL-based	MP2RAGE+FLAIR	0.72 ± 0.07	0.69	0.79	0.73
		MP2RAGE	0.71 ± 0.08	0.68	0.78	0.72
		FLAIR	0.66 ± 0.09	0.59	0.79	0.67
MindGlide	DL-based	MP2RAGE	0.38 ± 0.14	0.48	0.35	0.40
		FLAIR	0.44 ± 0.14	0.48	0.45	0.45
LGA	Conventional	MP2RAGE+FLAIR	0.33 ± 0.12	0.52	0.26	0.35

Values represent mean performance metrics calculated separately for each of the 20 test scans.

## Data Availability

The data related to this manuscript are available from the corresponding author upon reasonable request. Data requests should be directed to the corresponding author (assunta.dal-bianco@meduniwien.ac.at). Correspondence and requests for materials should be addressed to A.D.B.

## Supplementary Materials

The following supporting information can be downloaded at https://www.mdpi.com/article/10.3390/jimaging12100510/s1: Figure S1: Number of ground-truth lesions and lesions detected by nnUNet v2 and DeepMedic across lesion-volume categories for (A) MP2RAGE-only and (B) FLAIR-only input; Figure S2: Impact of lesion size filtering on segmentation performance (voxel level; *μ*—mean; *η*—median); Figure S3: Impact of lesion size filtering on lesion-level segmentation performance for nnUNet (*μ*—mean; *η*—median); Figure S4: Impact of lesion size filtering on lesion-level segmentation performance for DeepMedic (*μ*—mean. *η*—median); Table S1: Architectural and training characteristics of nnUNet v2 and DeepMedic; Table S2: Computational cost was estimated per network inference unit: a 128 × 128 × 128 patch for nnUNet v2 and a 45 × 45 × 45 high-resolution segment with the corresponding subsampled pathway input for DeepMedic; Table S3: Evaluation metrics; Table S4: Voxel-level evaluation metrics across all input configurations for nnUNet and DeepMedic; Table S5: Pairwise patient-level comparisons of voxel-level DSC between nnUNet and DeepMedic across the three MRI input configurations; Table S6: Pairwise patient-level comparisons of voxel-level DSC between MRI input configurations for nnUNet; Table S7: Pairwise patient-level comparisons of voxel-level DSC between MRI input configurations for DeepMedic; Table S8: Lesion-level detection performance across all configurations; Table S9: Lesion-level evaluation metrics across all configurations; Table S10: Voxel-level evaluation metrics across all configurations for nnUNet, MindGlide and LGA; Table S11: Generalized estimating equation (GEE) logistic regression analysis of lesion detection according to lesion volume; Table S12: Reassessment of apparent false-positive detections as lesions missed in the original ground truth.

## Abbreviations

The following abbreviations are used in this manuscript:

AI	Artificial Intelligence
ANTs	Advanced Normalization Tools
BET	Brain Extraction Tool
CNN	Convolutional Neural Network
CSF	Cerebrospinal Fluid
CVS	Central Vein Sign
DL	Deep Learning
DSC	Dice Similarity Coefficient
EDSS	Expanded Disability Status Scale
FDR	False Discovery Rate
FLAIR	Fluid-Attenuated Inversion Recovery
FLIRT	FMRIB’s Linear Image Registration Tool
FNR	False Negative Rate
FP	False Positive
FSL	FMRIB Software Library
GPU	Graphics Processing Unit
GT	Ground truth
IoU	Intersection over Union
LGA	Lesion Growth Algorithm
LST	Lesion Segmentation Tool
MCC	Matthews Correlation Coefficient
MRI	Magnetic Resonance Imaging
MS	Multiple Sclerosis
MP2RAGE	Magnetization-Prepared 2 Rapid Acquisition Gradient Echoes
PPV	Positive Predictive Value
PRL	Paramagnetic Rim Lesion
RRMS	Relapsing-Remitting Multiple Sclerosis
SPM	Statistical Parametric Mapping
SPMS	Secondary-Progressive Multiple Sclerosis
TE	Echo Time
TI	Inversion Time
TP	True Positive
TR	Repetition Time
WMH	White Matter Hyperintensity
