# Supplementary material for: Deep Learning-Based Multiple Sclerosis Lesion Segmentation at 7 Tesla: Comparison of Single- and Multi-Contrast MRI Inputs
Source: J Imaging. 2026 Oct 14;12(10):510. doi: 10.3390/jimaging12100510 (PMC13705961; doi:10.3390/jimaging12100510)
Supplement: Supplementary file 1 [file jimaging-12-00510-s001.zip › jimaging-4529391-supplementary.pdf]

Journal of Imaging

Supplementary Materials

Deep Learning-Based Multiple Sclerosis Lesion Segmentation at 7 Tesla: Comparison of Single- and Multi-Contrast MRI Inputs

Anna Petrova [1,2,3], Ivan Petrovic [1,2], Marc Berger [2,4], Yanniklas Kravutske [4], Joel Leinkauf [4], Rebeka Rumbak [1,2], Lukas Haider [3,4,5], Eva Niess [1], Bernhard Strasser [1], Wolfgang Bogner [1,6,7], Günther Grabner [8], Gabriel Bsteh [2,3], Thomas Berger [2,3], Paulus Rommer [2,3], Assunta Dal-Bianco [2,3,*] and Stanislav Motyka [1,6,7]

[1] High-Field MR Center, Department of Biomedical Imaging and Image-Guided Therapy, Medical University of Vienna, 1090 Vienna, Austria
[2] Department of Neurology, Medical University of Vienna, 1090 Vienna, Austria
[3] Comprehensive Center for Clinical Neurosciences & Mental Health, Medical University of Vienna, 1090 Vienna, Austria
[4] Division of Neuroradiology and Musculoskeletal Radiology, Department of Biomedical Imaging and Image-Guided Therapy, Medical University of Vienna, 1090 Vienna, Austria
[5] NMR Research Unit, Queen Square Multiple Sclerosis Centre, Queen Square Institute of Neurology, University College London, London WC1N 3BG, UK
[6] Comprehensive Center for AI in Medicine (CAIM), Medical University of Vienna, 1090 Vienna, Austria
[7] Christian Doppler Laboratory for MR Imaging Biomarkers (BIOMAK), Department of Biomedical Imaging and Image-guided Therapy, Medical University of Vienna, 1090 Vienna, Austria
[8] Department of Medical Engineering, Carinthia University of Applied Sciences, 9020 Klagenfurt, Austria
* Correspondence: assunta.dal-bianco@meduniwien.ac.at

Table S1. Architectural and training characteristics of nnUNet v2 and DeepMedic.

**Parameter**	**DeepMedic**	**nnUNet (3d full resolution)**
Architecture	Multi-scale 3D CNN	U-Net with plain convolutional encoder (PlainConvUNet)
Number of pathways/stages	2 pathways: normal-resolution and subsampled	6 encoder-decoder stages
Feature maps	Normal: 24, 24, 32, 32, 40, 40, 48, 48<br>Subsampled: 32, 32, 40, 40, 48, 48, 56, 56	32, 64, 128, 256, 320, 320
Convolutions per stage/pathway	8 convolutional layers per pathway	2 convolutions per encoder stage; 2 per decoder stage
Kernel size	3 × 3 × 3	3 × 3 × 3
Subsampling/downsampling	Subsampling factor 3 × 3 × 3	Stride 1×1×1 at the first stage. followed by 2 × 2 × 2
Input Type	3D patches	3D patches
Batch size	3	2
Patch size	Uses segments<br>For training [45 × 45 × 45]	128 × 128 × 128
Number epochs	80	1000
Subepochs per Epoch	10	-
Learning rate (initial)	0.0002	0.01
Optimizer	Adam	Adam
Loss Function	Dice loss (0.7) + Cross Entropy (0.3)	Dice loss
Activation Function	PreLU	LeakyReLU
Training strategy	Manually configured based on original framework recommendations	Automatically configured by nnUNet

Table S2. Computational cost was estimated per network inference unit: a 128 × 128 × 128 patch for nnUNet v2 and a 45 × 45 × 45 high-resolution segment with the corresponding subsampled pathway input for DeepMedic.

Model	Input	Trainable parameters	FLOPs/ inference unit	Inference time / scan	Peak observed GPU memory
nnUNet v2	Single contrast	31.196 M	2456.799 GFLOPs/128 × 128 × 128 patch	87.5 s	~3.49 GB
	MP2RAGE+FLAIR	31.196 M	2471.295 GFLOPs/128 × 128 × 128 patch	87.5 s	~3.49 GB
DeepMedic	Single contrast	1.004 M	40.642 GFLOPs/inference segment	30.0 s	~31.2 GB
	MP2RAGE+FLAIR	1.006 M	40.769 GFLOPs/inference segment	30.0 s	~31.2 GB

Table S3. Evaluation metrics.

Metrics	Formula
Dice Similarity Coefficient (DSC) / F1 score	$DSC = \frac{2 \times TP}{2 \times TP + FP + FN}$
Intersection Over Union (IoU)	$IoU = \frac{TP}{TP + FP + FN}$
Sensitivity (Recall)	$Recall = \frac{TP}{TP + FN}$
Specificity	$Specificity = \frac{TN}{TN + FP}$
False Negative Ratio (FNR)	$FNR = \frac{FN}{FN + TP}$
False Discovery Rate (FDR)	$FDR = \frac{FP}{FP + TP}$
Positive Predictive Value (PPV/Precision)	$PPV = \frac{TP}{TP + FP}$
Matthew's Correlation Coefficient (MCC)	$MCC = \frac{\left((TN * TP) - (FN * FP)\right)}{\sqrt{\left((TP + FP) * (TP + FN) * (TN + FP) * (TN + FN)\right)}}$

Table S4. Voxel-level evaluation metrics across all input configurations for nnUNetand DeepMedic.

Metric	FLAIR+MP2RAGE	MP2RAGE	FLAIR
Sensitivity (Recall)			
nnUNet	0.69 ± 0.14	0.68 ± 0.15	0.59 ± 0.14
DeepMedic	0.76 ± 0.11	0.75 ± 0.11	0.70 ± 0.14
False Negative Rate (FNR)			
nnUNet	0.31 ± 0.14	0.32 ± 0.15	0.41 ± 0.14
DeepMedic	0.24 ± 0.11	0.25 ± 0.11	0.30 ± 0.14
Positive Predictive Value (PPV)			
nnUNet	0.79 ± 0.12	0.78 ± 0.12	0.79 ± 0.12
DeepMedic	0.70 ± 0.13	0.70 ± 0.12	0.63 ± 0.13
False Discovery Rate (FDR)			
nnUNet	0.21 ± 0.12	0.22 ± 0.12	0.21 ± 0.12
DeepMedic	0.30 ± 0.13	0.30 ± 0.12	0.37 ± 0.13
Dice Similarity Coefficient (DSC)			
nnUNet	0.72 ± 0.07	0.71 ± 0.08	0.66 ± 0.09
DeepMedic	0.71 ± 0.06	0.71 ± 0.06	0.64 ± 0.07
Intersection Over Union (IoU)			

nnUNet	0.56 ± 0.09	0.55 ± 0.10	0.50 ± 0.10
DeepMedic	0.56 ± 0.07	0.55 ± 0.07	0.47 ± 0.08
Matthew's Correlation Coefficient (MCC)			
nnUNet	0.73 ± 0.07	0.72 ± 0.08	0.67 ± 0.08
DeepMedic	0.72 ± 0.06	0.72 ± 0.05	0.65 ± 0.06

Values represented as mean ± standard deviation (n = 20 scans from 11 patients).

Table S5. Pairwise patient-level comparisons of voxel-level DSC between nnUNet and DeepMedic across the three MRI input configurations. Raw p-values were obtained using two-sided Wilcoxon signed-rank tests. P-values were adjusted within the predefined family of comparisons using the Benjamini-Hochberg (BH) and Holm procedures. Effect sizes are reported as rank-biserial correlations and standardized paired effect sizes (d_z). Post hoc achieved power was estimated at $\alpha = 0.05$ as described in the Statistical Analysis section and is reported descriptively.

Comparison	Wilcoxon W	Raw p-value	BH-adjusted p-value	Holm-adjusted p-value	Rank-biserial r	d_z	Post hoc power
nnUNet vs DeepMedic, FLAIR+MP2RAGE	24.0	0.4648	0.6377	0.9297	0.2727	0.1745	7.1%
nnUNet vs DeepMedic, MP2RAGE	27.0	0.6377	0.6377	0.9297	0.1818	0.0541	4.4%
nnUNet vs DeepMedic, FLAIR	15.0	0.1230	0.3691	0.3691	0.5455	0.4802	26.5%

**All comparisons were based on n = 11 independent patients. Post hoc power estimates are descriptive and should be interpreted together with the corresponding effect sizes and p-values.*

Table S6. Pairwise patient-level comparisons of voxel-level DSC between MRI input configurations for nnUNet. Raw p-values were obtained using two-sided Wilcoxon signed-rank tests. P-values were adjusted within the predefined family of comparisons using the Benjamini-Hochberg (BH) and Holm procedures. Effect sizes are reported as rank-biserial correlations and standardized paired effect sizes (d_z). Post hoc achieved power was estimated at $\alpha = 0.05$ and is reported descriptively.

Comparison	Wilcoxon W	Raw p-value	BH-adjusted p-value	Holm-adjusted p-value	Rank-biserial r	d_z	Post hoc power
FLAIR+MP2RAGE vs MP2RAGE	17.0	0.1748	0.1748	0.1748	0.4848	0.3978	19.4%
FLAIR+MP2RAGE vs FLAIR	0.0	0.00097	0.00292	0.0029	1.0	1.3580	97.1%
MP2RAGE vs FLAIR	5.0	0.00976	0.0146	0.0195	0.8484	0.9537	77.1%

**All comparisons were based on n = 11 independent patients. Post hoc power estimates are descriptive and should be interpreted together with the corresponding effect sizes and p-values.*

Table S7. Pairwise patient-level comparisons of voxel-level DSC between MRI input configurations for DeepMedic. Raw p-values were obtained using two-sided Wilcoxon signed-rank tests. P-values were adjusted within the predefined family of comparisons using the Benjamini–Hochberg (BH) and Holm procedures. Effect sizes are reported as rank-biserial correlations and standardized paired effect sizes (d_z). Post hoc achieved power was estimated at $\alpha = 0.05$ and is reported descriptively.

Comparison	Wilcoxon W	Raw p-value	BH-adjusted p-value	Holm-adjusted p-value	Rank-biserial r	d_z	Post hoc power
FLAIR+MP2RAGE vs MP2RAGE	19.0	0.2402	0.2402	0.2402	0.4242	0.3165	13.6%

Comparison	Wilcoxon W	Raw p-value	BH-adjusted p-value	Holm-adjusted p-value	Rank-biserial r	d_z	Post hoc power
FLAIR+MP2RAGE vs FLAIR	0.0	0.00097	0.00146	0.0029	1.0000	1.6638	99.7%
MP2RAGE vs FLAIR	0.0	0.00097	0.00146	0.0029	1.0000	1.4211	98.1%

**All comparisons were based on n = 11 independent patients. Post hoc power estimates are descriptive and should be interpreted together with the corresponding effect sizes and p-values.*

Table S8. Lesion-level detection performance across all configurations.

Case	Model	Thr		FLAIR+MP2RAGE			MP2RAGE			FLAIR		
			GT	TP	FN	FP	TP	FN	FP	TP	FN	FP
#1	nnUNet	1%	35	29	6	8	28	7	5	29	6	8
		30%		27	8		27	8		28	7	
	DeepMedic	1%		30	5	19	31	4	22	29	6	34
		30%		29	6		30	5		26	9	
#2	nnUNet	1%	47	35	12	3	33	14	2	33	14	4
		30%		31	16		30	17		28	19	
	DeepMedic	1%		41	6	10	38	9	14	34	13	35
		30%		37	10		35	12		29	18	
#3	nnUNet	1%	54	35	19	4	31	23	3	36	18	9
		30%		35	19		30	24		31	23	
	DeepMedic	1%		51	3	34	49	5	23	50	4	43
		30%		50	4		48	6		49	5	
#4	nnUNet	1%	64	47	17	4	43	21	6	39	25	5
		30%		43	21		41	23		38	26	
	DeepMedic	1%		56	8	25	54	10	30	55	9	36
		30%		54	10		53	11		53	11	
#5	nnUNet	1%	28	24	4	11	24	4	9	23	5	12
		30%		24	4		21	7		22	6	
	DeepMedic	1%		26	2	25	25	3	18	24	4	40
		30%		26	2		24	2		24	4	
#6	nnUNet	1%	76	26	50	2	33	43	1	28	48	9
		30%		19	57		27	49		22	54	
	DeepMedic	1%		50	26	38	53	23	19	45	31	43
		30%		36	40		45	31		39	37	
#7	nnUNet	1%	49	34	15	5	33	16	3	39	10	21
		30%		29	20		30	19		35	14	
	DeepMedic	1%		41	8	47	41	8	32	43	6	42
		30%		35	14		38	11		43	6	
#8	nnUNet	1%	89	70	19	14	59	30	8	60	29	7
		30%		61	28		56	33		53	36	
	DeepMedic	1%		80	9	43	80	9	36	69	20	38
		30%		70	19		70	19		62	27	
#9	nnUNet	1%	45	40	5	9	42	3	9	28	17	5
		30%		40	5		42	3		23	22	
	DeepMedic	1%		43	2	20	44	1	15	36	9	23
		30%		42	3		43	2		34	11	
#10	nnUNet	1%	53	43	10	5	46	7	4	29	24	0
		30%		41	12		43	10		27	26	
	DeepMedic	1%		46	7	8	49	4	12	41	12	17

		30%		43	10		45	8		37	16	
#11	nnUNet	1%	65	44	21	8	40	25	12	39	26	6
		30%		43	22		40	25		35	30	
	DeepMedic	1%		45	20	20	46	19	22	40	25	31
		30%		45	20		44	21		38	27	
#12	nnUNet	1%	57	50	7	12	51	6	10	40	17	7
		30%		48	9		49	8		37	20	
	DeepMedic	1%		55	2	15	54	3	18	46	11	25
		30%		53	4		51	6		45	12	
#13	nnUNet	1%	52	36	16	10	37	15	7	28	24	6
		30%		33	19		35	17		26	26	
	DeepMedic	1%		41	11	14	42	10	15	33	19	25
		30%		38	14		41	11		32	20	
#14	nnUNet	1%	15	11	4	2	11	4	0	11	4	2
		30%		10	5		10	5		10	5	
	DeepMedic	1%		13	2	4	12	3	12	13	2	19
		30%		11	4		11	4		12	3	
#15	nnUNet	1%	8	7	1	1	7	1	0	7	1	0
		30%		7	1		7	1		6	2	
	DeepMedic	1%		7	1	14	7	1	10	7	1	19
		30%		7	1		7	1		7	1	
#16	nnUNet	1%	21	7	14	0	9	12	0	6	15	0
		30%		6	15		8	13		6	15	
	DeepMedic	1%		11	10	5	11	10	4	10	11	4
		30%		9	12		9	12		7	14	
#17	nnUNet	1%	20	6	14	1	7	13	1	4	16	0
		30%		5	15		7	13		4	16	
	DeepMedic	1%		11	9	6	10	10	7	8	12	11
		30%		9	11		9	11		8	12	
#18	nnUNet	1%	53	37	16	20	38	15	26	26	27	5
		30%		35	18		34	19		20	33	
	DeepMedic	1%		41	12	39	39	14	32	26	27	16
		30%		36	17		33	20		21	32	
#19	nnUNet	1%	67	39	28	7	41	26	10	26	41	2
		30%		36	31		38	29		20	47	
	DeepMedic	1%		43	24	27	44	23	19	25	41	20
		30%		35	32		39	28		20	47	
#20	nnUNet	1%	19	19	0	5	19	0	3	15	4	7
		30%		18	1		19	0		15	4	
	DeepMedic	1%		19	0	23	19	0	18	17	2	24
		30%		19	0		19	0		16	3	

GT: Ground Truth; TP: True Positives; FN: False Negatives; FP: False Positives; Thr: Threshold.

Table S9. Lesion-level evaluation metrics across all configurations.

Case	Model	Thr	FLAIR+MP2RAGE			MP2RAGE			FLAIR		
			F1	PPV	Sens	F1	PPV	Sens	F1	PPV	Sens
#1	nnUNet	1%	0.81	0.78	0.83	0.82	0.85	0.80	0.81	0.78	0.83
		30%	0.77	0.77	0.77	0.81	0.84	0.77	0.79	0.78	0.80
	DeepMedic	1%	0.84	0.80	0.87	0.71	0.59	0.89	0.59	0.46	0.83
		30%	0.79	0.79	0.79	0.69	0.57	0.86	0.55	0.43	0.74
#2	nnUNet	1%	0.82	0.92	0.74	0.81	0.94	0.70	0.79	0.89	0.70
		30%	0.77	0.91	0.66	0.76	0.94	0.64	0.71	0.88	0.60
	DeepMedic	1%	0.71	0.61	0.86	0.77	0.73	0.81	0.59	0.49	0.72
		30%	0.70	0.60	0.83	0.73	0.71	0.74	0.52	0.45	0.62
#3	nnUNet	1%	0.75	0.90	0.65	0.71	0.91	0.57	0.73	0.80	0.67
		30%	0.75	0.90	0.65	0.69	0.91	0.56	0.66	0.78	0.57
	DeepMedic	1%	0.73	0.60	0.94	0.78	0.68	0.91	0.68	0.54	0.93
		30%	0.73	0.60	0.93	0.77	0.68	0.89	0.67	0.53	0.91
#4	nnUNet	1%	0.82	0.92	0.73	0.76	0.88	0.67	0.72	0.89	0.61
		30%	0.78	0.92	0.67	0.74	0.87	0.64	0.71	0.88	0.59
	DeepMedic	1%	0.77	0.69	0.88	0.73	0.64	0.84	0.71	0.60	0.86
		30%	0.76	0.68	0.84	0.72	0.64	0.83	0.69	0.60	0.83
#5	nnUNet	1%	0.76	0.69	0.86	0.79	0.73	0.86	0.73	0.66	0.82
		30%	0.76	0.69	0.86	0.72	0.70	0.75	0.71	0.65	0.79
	DeepMedic	1%	0.66	0.51	0.93	0.70	0.58	0.89	0.52	0.38	0.86
		30%	0.66	0.51	0.93	0.71	0.57	0.92	0.52	0.38	0.86
#6	nnUNet	1%	0.50	0.92	0.34	0.60	0.97	0.43	0.49	0.76	0.37
		30%	0.39	0.91	0.25	0.52	0.96	0.36	0.41	0.71	0.29
	DeepMedic	1%	0.61	0.57	0.66	0.72	0.74	0.70	0.55	0.51	0.59
		30%	0.48	0.49	0.47	0.64	0.70	0.59	0.49	0.48	0.51
#7	nnUNet	1%	0.77	0.87	0.69	0.78	0.92	0.67	0.72	0.65	0.80
		30%	0.70	0.85	0.59	0.73	0.91	0.61	0.68	0.63	0.71
	DeepMedic	1%	0.60	0.47	0.84	0.67	0.56	0.84	0.64	0.51	0.88
		30%	0.53	0.43	0.71	0.64	0.54	0.78	0.64	0.51	0.88
#8	nnUNet	1%	0.84	0.81	0.79	0.86	0.84	0.66	0.77	0.85	0.67
		30%	0.82	0.80	0.69	0.85	0.83	0.63	0.73	0.84	0.60
	DeepMedic	1%	0.87	0.79	0.90	0.84	0.75	0.90	0.72	0.65	0.78
		30%	0.85	0.78	0.79	0.81	0.74	0.79	0.71	0.64	0.70
#9	nnUNet	1%	0.85	0.82	0.89	0.88	0.82	0.93	0.72	0.85	0.62
		30%	0.85	0.82	0.89	0.88	0.82	0.93	0.63	0.82	0.51
	DeepMedic	1%	0.80	0.68	0.96	0.85	0.75	0.98	0.69	0.61	0.8
		30%	0.79	0.68	0.93	0.84	0.74	0.96	0.68	0.60	0.76
#10	nnUNet	1%	0.85	0.90	0.81	0.89	0.92	0.87	0.71	1	0.55
		30%	0.83	0.89	0.77	0.86	0.92	0.81	0.68	1	0.51
	DeepMedic	1%	0.86	0.85	0.87	0.86	0.80	0.92	0.74	0.71	0.77
		30%	0.83	0.84	0.81	0.82	0.79	0.85	0.69	0.69	0.70
#11	nnUNet	1%	0.75	0.85	0.68	0.68	0.77	0.62	0.71	0.87	0.60
		30%	0.74	0.84	0.66	0.68	0.77	0.62	0.66	0.85	0.54
	DeepMedic	1%	0.69	0.69	0.69	0.69	0.68	0.71	0.59	0.56	0.62
		30%	0.69	0.69	0.69	0.67	0.67	0.68	0.57	0.55	0.58
#12	nnUNet	1%	0.84	0.81	0.88	0.86	0.84	0.89	0.77	0.85	0.70
		30%	0.82	0.80	0.84	0.85	0.83	0.86	0.73	0.84	0.65
	DeepMedic	1%	0.87	0.79	0.96	0.84	0.75	0.95	0.72	0.65	0.81

		30%	0.85	0.78	0.93	0.81	0.74	0.89	0.71	0.64	0.79
#13	nnUNet	1%	0.74	0.78	0.69	0.77	0.84	0.71	0.65	0.82	0.54
		30%	0.70	0.77	0.63	0.75	0.83	0.67	0.62	0.81	0.50
	DeepMedic	1%	0.77	0.75	0.79	0.77	0.74	0.81	0.60	0.57	0.63
		30%	0.75	0.75	0.75	0.76	0.73	0.79	0.59	0.56	0.62
#14	nnUNet	1%	0.79	0.85	0.73	0.85	1	0.73	0.79	0.85	0.73
		30%	0.74	0.83	0.67	0.80	1	0.67	0.74	0.83	0.67
	DeepMedic	1%	0.81	0.77	0.87	0.61	0.50	0.8	0.55	0.41	0.87
		30%	0.73	0.73	0.73	0.58	0.48	0.73	0.52	0.39	0.8
#15	nnUNet	1%	0.88	0.88	0.88	0.93	1	0.88	0.93	1	0.88
		30%	0.88	0.88	0.88	0.93	1	0.88	0.86	1	0.75
	DeepMedic	1%	0.48	0.33	0.88	0.56	0.41	0.88	0.41	0.27	0.88
		30%	0.48	0.33	0.88	0.56	0.41	0.88	0.41	0.27	0.88
#16	nnUNet	1%	0.50	1	0.33	0.60	1	0.43	0.44	1	0.29
		30%	0.44	1	0.29	0.55	1	0.38	0.44	1	0.29
	DeepMedic	1%	0.60	0.69	0.52	0.61	0.73	0.52	0.57	0.71	0.48
		30%	0.51	0.64	0.43	0.53	0.63	0.43	0.44	0.64	0.33
#17	nnUNet	1%	0.44	0.86	0.3	0.50	0.88	0.35	0.33	1	0.2
		30%	0.39	0.83	0.25	0.50	0.88	0.35	0.33	1	0.2
	DeepMedic	1%	0.60	0.65	0.55	0.54	0.59	0.5	0.41	0.42	0.4
		30%	0.51	0.60	0.45	0.50	0.56	0.45	0.41	0.42	0.4
#18	nnUNet	1%	0.67	0.65	0.70	0.65	0.59	0.72	0.62	0.84	0.49
		30%	0.65	0.64	0.66	0.60	0.57	0.64	0.51	0.80	0.38
	DeepMedic	1%	0.62	0.51	0.77	0.63	0.55	0.74	0.55	0.62	0.49
		30%	0.56	0.48	0.68	0.56	0.51	0.62	0.47	0.57	0.40
#19	nnUNet	1%	0.69	0.85	0.58	0.70	0.80	0.61	0.55	0.93	0.39
		30%	0.65	0.84	0.54	0.66	0.79	0.57	0.44	0.91	0.30
	DeepMedic	1%	0.63	0.61	0.64	0.68	0.70	0.66	0.46	0.57	0.39
		30%	0.54	0.57	0.52	0.62	0.67	0.58	0.37	0.50	0.30
#20	nnUNet	1%	0.88	0.79	1.00	0.93	0.86	1	0.73	0.68	0.79
		30%	0.86	0.78	0.95	0.93	0.86	1	0.73	0.68	0.79
	DeepMedic	1%	0.62	0.45	1	0.68	0.51	1	0.57	0.42	0.89
		30%	0.62	0.45	1	0.68	0.51	1	0.54	0.40	0.84

F1: F1 score; PPV: Positive Predictive Value; Sens: Sensitivity.

Table S10. Voxel-level evaluation metrics across all configurations for nnUNet. MindGlide and LGA.

Metric	FLAIR+MP2RAGE	MP2RAGE	FLAIR
Sensitivity (Recall)			
nnUNet	0.69 ± 0.14	0.68 ± 0.15	0.59 ± 0.14
MindGlide	-	0.48 ± 0.15	0.48 ± 0.15
LGA	0.52 ± 0.14	-	-
False Negative Rate (FNR)			
nnUNet	0.31 ± 0.14	0.32 ± 0.15	0.41 ± 0.14
MindGlide	-	0.52 ± 0.15	0.52 ± 0.15
LGA	0.48 ± 0.14	-	-
Positive Predictive Value (PPV)			
nnUNet	0.79 ± 0.12	0.78 ± 0.12	0.79 ± 0.12
MindGlide	-	0.35 ± 0.16	0.45 ± 0.18
LGA	0.26 ± 0.12	-	-

False Discovery Rate (FDR)			
nnUNet	0.21 ± 0.12	0.22 ± 0.12	0.21 ± 0.12
MindGlide	-	0.65 ± 0.16	0.55 ± 0.18
LGA	0.74 ± 0.12	-	-
Dice Similarity Coefficient (DSC)			
nnUNet	0.72 ± 0.07	0.71 ± 0.08	0.66 ± 0.09
MindGlide	-	0.38 ± 0.14	0.44 ± 0.14
LGA	0.33 ± 0.12	-	-
Intersection Over Union (IoU)			
nnUNet	0.56 ± 0.09	0.55 ± 0.10	0.50 ± 0.10
MindGlide	-	0.25 ± 0.11	0.29 ± 0.11
LGA	0.20 ± 0.09	-	-
Matthew's Correlation Coefficient (MCC)			
nnUNet	0.73 ± 0.07	0.72 ± 0.08	0.67 ± 0.08
MindGlide	-	0.40 ± 0.14	0.45 ± 0.13
LGA	0.35 ± 0.11	-	-

Values represented as mean ± standard deviation (n = 20 scans from 11 patients).

Table S11. Generalized estimating equation (GEE) logistic regression analysis of lesion detection according to lesion volume. Patient ID was used as the clustering variable. Lesion detection was defined as the presence of at least one predicted voxel overlapping the ground-truth lesion. For the continuous analysis. the natural logarithm of lesion volume was used as the predictor and models were fitted separately for nnUNet v2 and DeepMedic using combined MP2RAGE+FLAIR input. The categorical analysis compared lesions measuring 50–200 mm³ with lesions measuring 5–20 mm³ (reference category) separately for each architecture and input configuration. OR. odds ratio; CI. confidence interval.

Model	Input	Analysis	OR	95% CI	p-value
nnUNet	MP2RAGE+FLAIR	ln (lesion volume)	1.92	1.72–2.14	<0.001
DeepMedic	MP2RAGE+FLAIR	ln (lesion volume)	2.33	1.97–2.74	<0.001
	MP2RAGE+FLAIR	50–200 vs. 5–20 mm³	3.46	2.68–4.46	<0.001
nnUNet	MP2RAGE	50–200 vs. 5–20 mm³	3.96	3.00–5.23	<0.001
	FLAIR	50–200 vs. 5–20 mm³	4.54	3.33–6.17	<0.001
	MP2RAGE+FLAIR	50–200 vs. 5–20 mm³	7.88	4.47–13.89	<0.001
DeepMedic	MP2RAGE	50–200 vs. 5–20 mm³	7.94	4.77–13.22	<0.001
	FLAIR	50–200 vs. 5–20 mm³	9.95	6.21–15.92	<0.001

Table S12. Reassessment of apparent false-positive detections as lesions missed in the original ground truth.

Model	Input	Apparent FP reclassified as true lesions
	MP2RAGE+FLAIR	63%
nnUNet	MP2RAGE*	65%
	FLAIR*	59%
	MP2RAGE+FLAIR	60%
DeepMedic	MP2RAGE*	55%
	FLAIR*	37%

*The reassessment of the single-contrast configurations was performed by one expert and should therefore be considered exploratory.

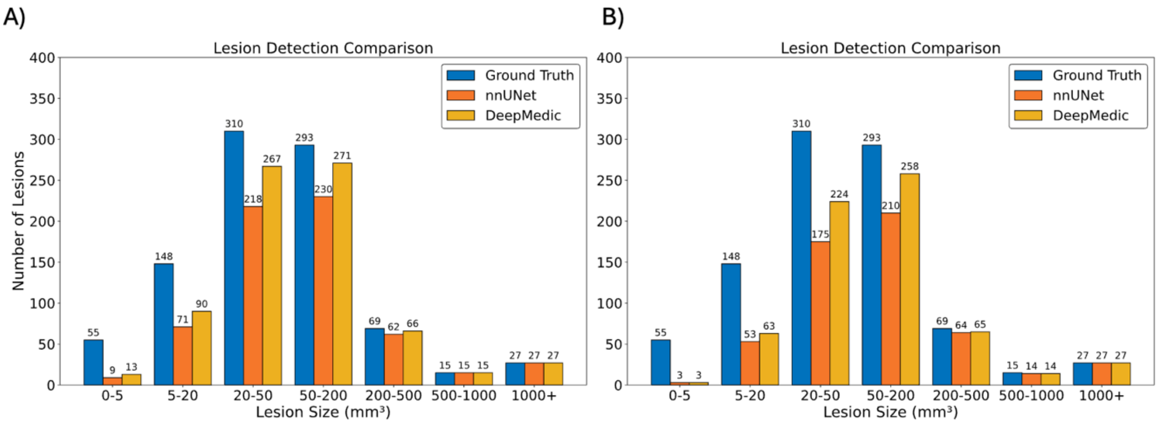

Figure S1. Number of ground-truth lesions and lesions detected by nnUNet v2 and DeepMedic across lesion-volume categories for (**A**) MP2RAGE-only and (**B**) FLAIR-only input.

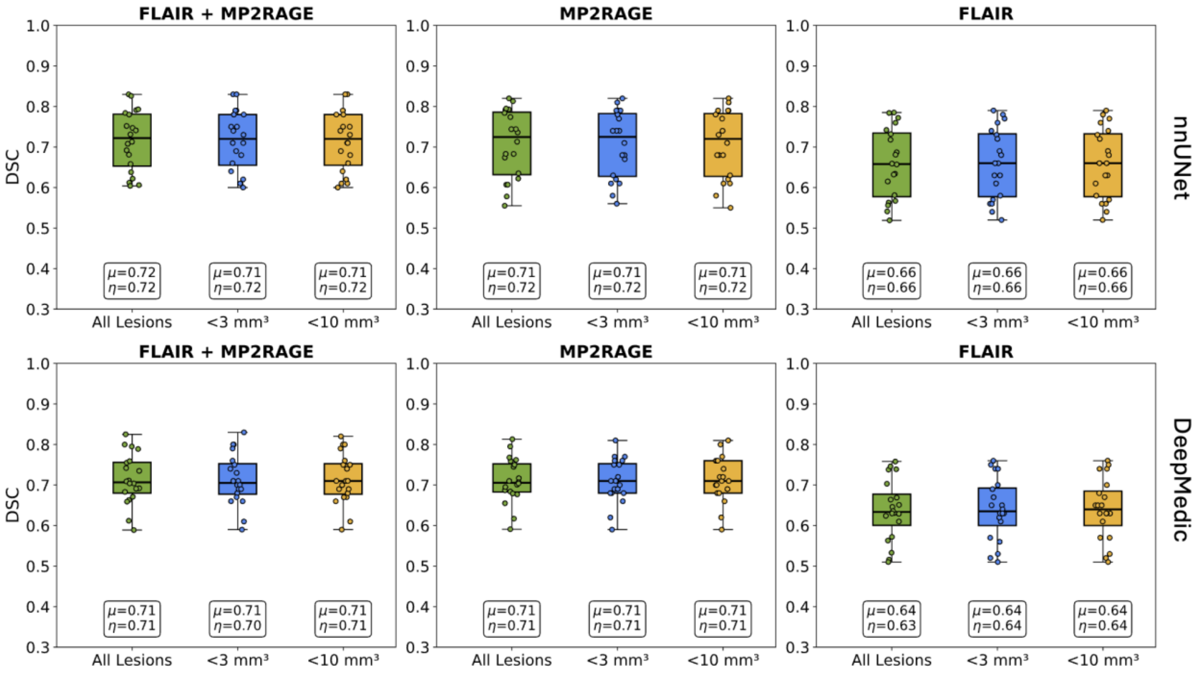

Figure S2. Impact of lesion size filtering on segmentation performance (voxel level. μ—mean. η—median).

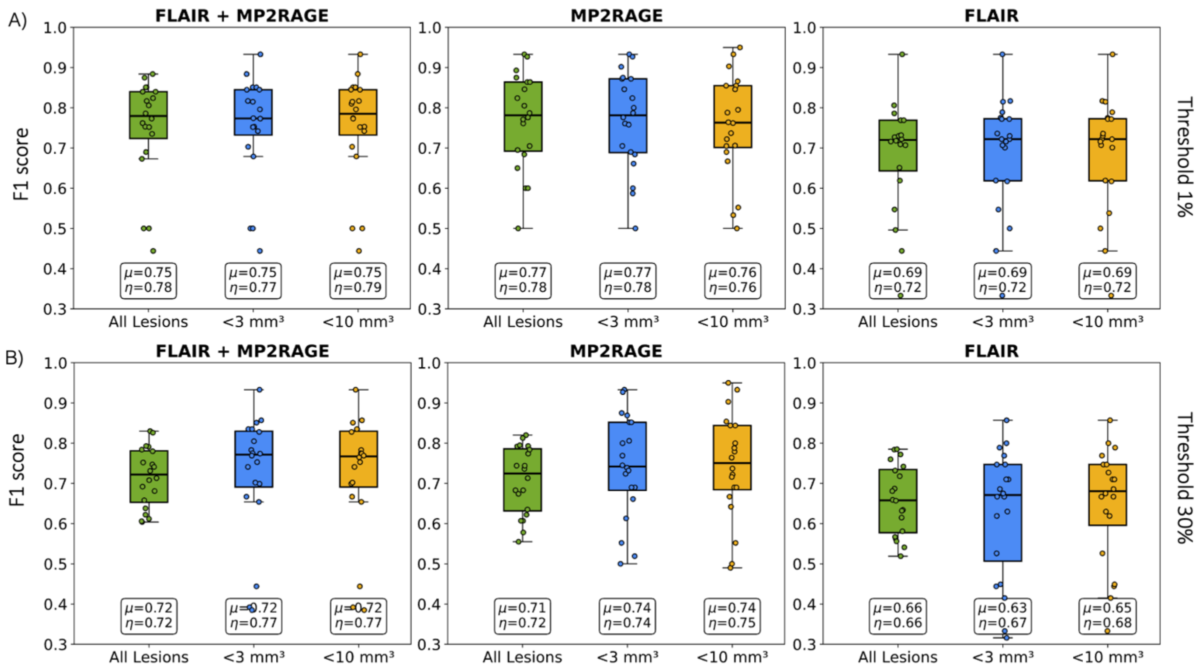

Figure S3. Impact of lesion size filtering on lesion-level segmentation performance for nnUNet (μ—mean. η—median).

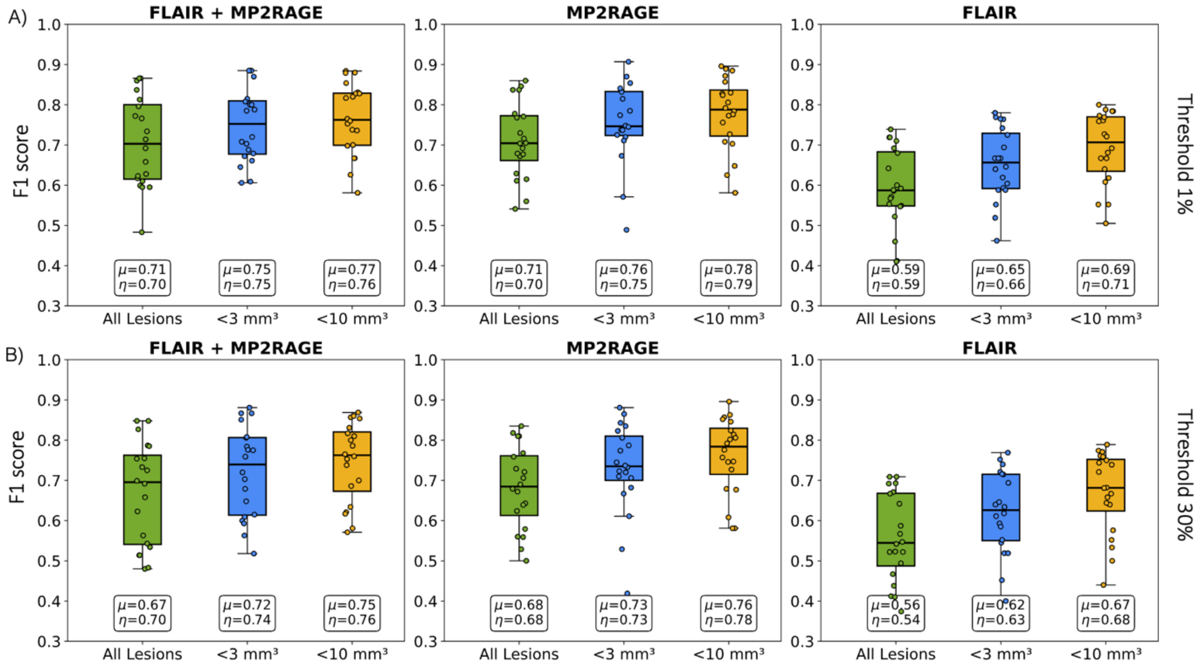

Figure S4. Impact of lesion size filtering on lesion-level segmentation performance for DeepMedic (μ—mean. η—median).
